# From randomness to recognition: modeling the evolution of DNA sequence information during enrichment for binding

**DOI:** 10.1093/bioadv/vbag118

**Published:** 2026-04-26

**Authors:** Varun Maher, Daniel Martin, David Spetzler, Zhan-Gong Zhao, Heather O’Neill, Neal W Woodbury

**Affiliations:** Precision Medicine Target and Drug Discovery, Caris Life Sciences, 350 W. Washington St., 4th Floor, Tempe, AZ, 85288, United States; School of Molecular Sciences, Arizona State University, Bateman Physical Sciences Center (Room PSD 104), Tempe, AZ, 85287, United States; Center for Molecular Design and Biomimetics, Biodesign Institute, Arizona State University, 1001 S. McAllister Ave, Tempe, AZ, 85287, United States; Precision Medicine Target and Drug Discovery, Caris Life Sciences, 350 W. Washington St., 4th Floor, Tempe, AZ, 85288, United States; Precision Medicine Target and Drug Discovery, Caris Life Sciences, 350 W. Washington St., 4th Floor, Tempe, AZ, 85288, United States; Precision Medicine Target and Drug Discovery, Caris Life Sciences, 350 W. Washington St., 4th Floor, Tempe, AZ, 85288, United States; Precision Medicine Target and Drug Discovery, Caris Life Sciences, 350 W. Washington St., 4th Floor, Tempe, AZ, 85288, United States; School of Molecular Sciences, Arizona State University, Bateman Physical Sciences Center (Room PSD 104), Tempe, AZ, 85287, United States; Center for Molecular Design and Biomimetics, Biodesign Institute, Arizona State University, 1001 S. McAllister Ave, Tempe, AZ, 85287, United States

## Abstract

**Motivation:**

Enrichment of random nucleic acid libraries for binding to a target can result in a specific aptamer sequence, but quantitative models describing the detailed evolution of molecular information during such processes are still lacking.

**Results:**

A masked language model (MLM) trained on unlabeled DNA sequences after partial enrichment to multiple targets was used to create an encoder that generates latent space sequence representations encompassing the attributes of binding enriched libraries. Independent replicate enrichments against the same target converged to nearly identical latent representations despite containing no overlapping sequences, demonstrating that the representation captured the functional characteristics of the library specific to the target. The degree of divergence of enriched libraries from the original unenriched library strongly correlated with experimental binding, and classifier performance based on latent embeddings captured target specificity, including single amino acid differences. These results show that latent space models provide a quantitative measure of molecular information evolution during enrichment and can provide evidence of binding outcomes, offering both conceptual insight into the evolution of molecular information and practical strategies for designing more effective initial libraries and enrichment processes.

**Availability:**

The datasets and code are available on Zenodo: https://doi.org/10.5281/zenodo.14941815.

## Introduction

Methods for enriching random molecular libraries for function, such as binding to a specific target, are well known and widely used (Huang *et al*. 2019, Ito *et al*. 2023, Brown *et al*. 2024, Iqbal *et al*. 2025). However, the evolution of information during that process remains less well understood, in part due to the lack of models for monitoring how a particular enrichment protocol imprints functional information in molecular structures, particularly when starting from random libraries that are themselves sparse samples of a much larger set of possible molecular species. The ability to follow the evolution of this functional information during enrichment, independent of the specific set of structures that mediate it, would both provide new insight into the topology of functional molecular spaces as well as practical insight into the most efficient means of optimizing structure/function relationships.

A facile and well-known model system for exploring the evolution of information during enrichment is Systematic Evolution of Ligands by Exponential Enrichment (SELEX) which iteratively enriches a diverse library of nucleic acid sequences ($10^{8}$–$10^{15}$ unique species) for specific binding to a target of interest. Since its introduction (Ellington and Szostak 1990, Tuerk and Gold 1990), SELEX has been used to generate aptamers for diverse targets, including organic dyes (Ellington and Szostak 1990), small molecules (Ellington and Szostak 1992, Famulok and Szostak 1992, Lorsch and Szostak 1994), proteins (Kubik *et al*. 1994, Nieuwlandt *et al*. 1995, Chen *et al*. 1996, Dang and Jayasena 1996, Williams *et al*. 1997), tumor marker peptides (Santana-Viera *et al*. 2023), and complex targets such as cells or lysates from FFPE tissues (Meyer *et al*. 2013, Domenyuk *et al*. 2018, Hornung *et al*. 2020).

Recent advances in applying machine learning and natural language processing methods to macromolecules like proteins and nucleic acids have made it possible to create meaningful representations of sequences that incorporate functional capability. This in turn opens the door for developing new tools for measuring and characterizing the information content of molecules and functionally enriched molecular libraries, which should both provide new insights into the evolution of molecular information as well as suggesting practical strategies for library design and enrichment methodology.

Various computational approaches have been used to predict the binding of aptamer sequences, either directly in silico or from high-throughput SELEX data obtained during the enrichment process and have been the subject of several recent reviews (Sun *et al*. 2022, Lee *et al*. 2023, Kumar *et al*. 2024). Many of these approaches rely on identifying potential aptamer sequences by clustering enriched sequences based on similarity, searching for motifs and common n-mers, and scoring candidates on criteria such as binding affinity and secondary structure stability (Hoinka *et al*. 2012, Jiang *et al*. 2014, Caroli *et al*. 2016, 2020, Ishida *et al*. 2020). The use of machine learning and deep learning models effectively allows screening of large datasets to identify potential high-affinity candidates (Asif and Orenstein 2020, Zhang *et al*. 2021). Other examples include SMART-Aptamer (Song *et al*. 2020) and RaptRanker (Ishida *et al*. 2020), which rank aptamers based on sequence abundance, motif stability, and predicted structure.

In addition to identification, computational methods are increasingly used for potential aptamer sequence optimization. A few tools have been developed for secondary (2D) and tertiary (3D) structure prediction, such as Mfold (Zuker 2003) and RNAComposer (Biesiada *et al*. 2016), and these provide metrics that can be used in optimizing aptamer-target interactions at a molecular level. In addition, a few researchers are integrating molecular docking and molecular dynamics simulations into aptamer optimization protocols (Hiller *et al*. 2006, Zhou *et al*. 2015). Tools such as AutoDock (Trott and Olson 2010) and GROMACS (Abraham *et al*. 2015) assess the stability and binding energies of aptamer-target complexes, and this facilitates further optimization through iterative sequence modifications.

The approaches described above mostly focus on finding discrete motifs or other sequence patterns, often augmented by either structural predictions or binding predictions. The success of large language models, and their counterparts in the analysis of biological polymers (Abramson *et al*. 2024, Oikonomou *et al*. 2024), suggests that it should be possible to look at the sequence space of enriched libraries in a similar fashion, developing latent space representations of sequences related to enrichment towards binding to a particular target. Natural Language Processing (NLP) has shown promise in building biological sequence-based embeddings that can be used as latent space representations to integrate information for use in mapping the biological properties of the sequences themselves. Modern NLP works by building representations of words or sequences that capture their meaning relative to other words. This type of representation learning allows the exploration of features of raw data in an unlabeled fashion, uncovering complex associations that are otherwise difficult to identify. Biological sequences vectorized by representation learning have successfully been used in tasks such as structure prediction (Rives *et al*. 2021, Abramson *et al*. 2024) and function association (Asgari and Mofrad 2015) using similarity and differences within these sequences in representation space. Deep learning approaches with raw protein sequences have shown promise in creating models and embeddings through representation learning which are then used to extract features for predictive tasks such as cell compartmentalization of proteins and predicting biological properties of engineered sequences (Yang *et al*. 2018). Additionally, learned representations offer an opportunity to visualize groups of sequences with similar features in latent space after dimension reduction. In previous sequence library analysis using dimension reduction, a variational autoencoder was used to create an embedding for in-silico aptamer generation where simulated sequence data was found to be distributed in latent space based on motif information (Iwano *et al*. 2022). Sequence embeddings were created using two selection datasets against separate targets and this was then used to successfully generate truncated sequences also capable of binding the targets. More recently, the use of latent space representation has been extended to the development of a diffusion model for generative prediction (Wang *et al*. 2024).

Here, latent space sequence representations based on data from sequencing SELEX enriched DNA libraries (Selection targets are listed in Table 1) are created using a masked language model with bidirectional Long Short-Term Memory architecture, a type of recurrent neural network. This approach is similar to one that has been used previously to successfully generate protein sequence embeddings (Bepler and Berger 2021). These latent space sequence representations are then interrogated for their ability to effectively capture information that is correlated with biological properties of the sequences, such as binding affinity to the enrichment target and specificity for one target vs another in targets with varying degrees of similarity. A key aspect of this approach is its ability to obtain target-specific information simply by characterizing the enriched sequences, without explicitly incorporating binding or target information during the training of the model. Using this approach, several fundamental questions can be explored. Do two independent enrichments using the same target result in equivalent latent space representations? Are the latent space representations specific enough to differentiate one target from another? How similar can two targets be and still create different latent space representations? Is there enough target-specific information in the latent space representation to predict binding to the target? In addition, this study also provides a foundation for using these models both as a predictive tool that can guide the SELEX or other enrichment approaches by measuring target-specific information content during the enrichment process and as a means of exploring the molecular recognition relationship between target structure and sequence content in enriched libraries.

**Table 1:** Target peptides and protein domains used.

Selection target	Target type	Replicates	Sequence	Length (aa)
WT Peptide 1	Peptide	3	DLVFLLDGSSRLSEAEFEVLKAFVVDMME**R**	30
Mut Peptide 1	Peptide	3	DLVFLLDGSSRLSEAEFEVLKAFVVDMME**Q**	30
WT Peptide 2	Peptide	3	VLKAFVVDMME**R**LRISQKWVRVAVVEYHDG	30
Mut Peptide 2	Peptide	3	VLKAFVVDMME**Q**LRISQKWVRVAVVEYHDG	30
vWF-A1-WT	Domain	3	Refer to supplementary reagent list	207
vWF-A1-2B	Domain	3	Refer to supplementary reagent list	207

Peptides were synthesized to >98% purity. Bold text indicates the R1306Q mutation site.

## Methods and materials

**Figure 1:**
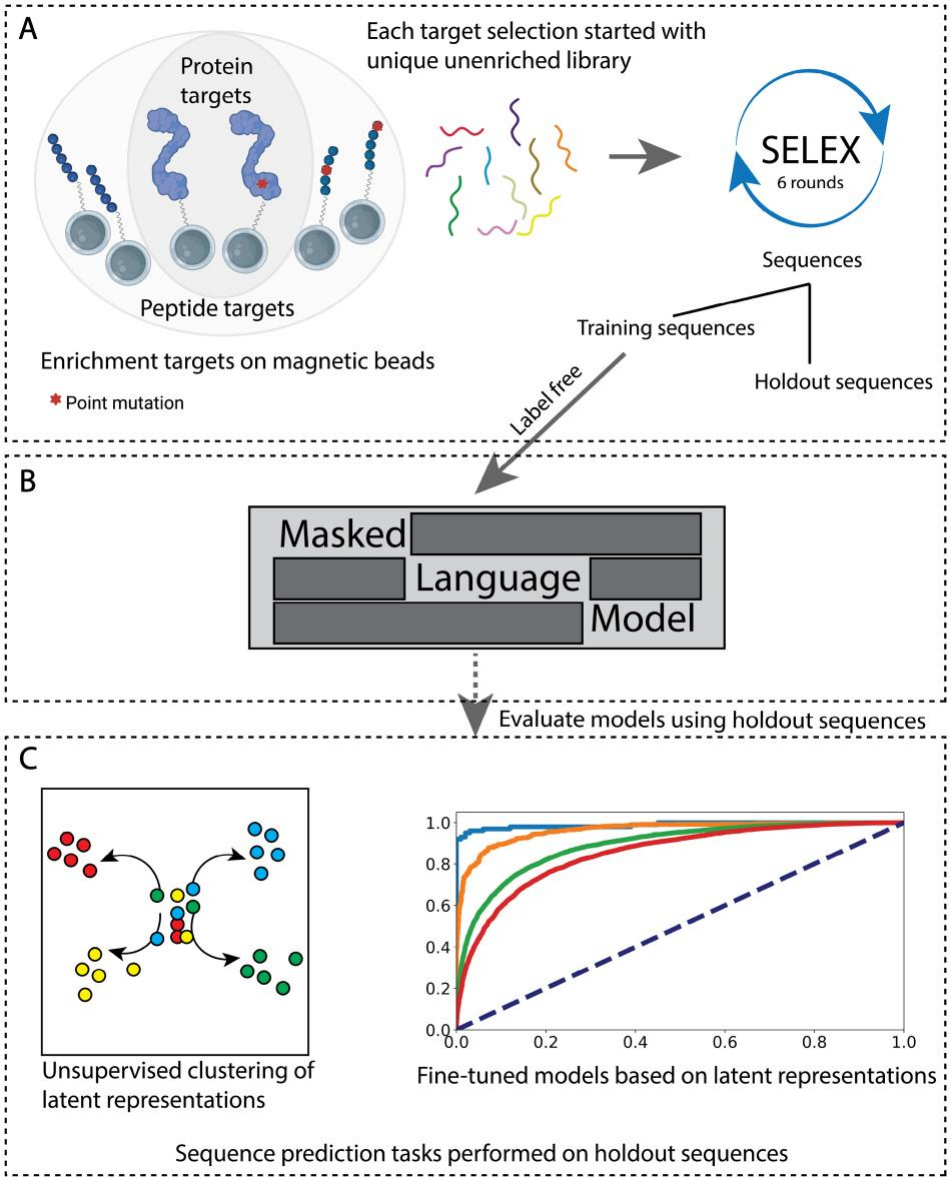
Overall analysis workflow. (A) Individual enrichments performed against protein and overlapping peptide targets, all started with unique unenriched libraries. (B) Label free sequences from selections used with a masked language model to create a pre-trained sequence model. This label free model can be used to (C) create latent space representations of holdout sequences to use in analysis of trends in information content and to train predictive classifiers mapping target labels to DNA aptamer sequences. Created in BioRender. Maher, V. (2026) https://BioRender.com/vtpeouf

The general workflow used in this study is shown in Fig. 1. All peptides were synthesized by LifeTein (Hillsborough, New Jersey, USA), with products purified by HPLC to guarantee >98% purity. The two recombinant domains were purchased from IPAtherapeutics (Victoria, BC, Canada). All custom oligonucleotides were synthesized and purchased from Integrated DNA Technologies (Coralville, Iowa, USA). Pools of biotinylated unenriched sequences consisted of a random 35 nucleotide variable region flanked by 2 constant primer regions, a 20-nucleotide forward primer binding region and a 15-nucleotide reverse binding region. The sense primer was biotinylated while the anti-sense primer was functionalized with a dibenzocyclooctyne (DBCO) moiety separated from the primer region via a poly A tail (35 nucleotides in length) and 2 internal 18 carbon spacers to reduce interaction between the sequences and the azide beads used as a solid support. The sequence format of the unenriched pools and the primers are provided in Supplementary Table S1. Supplementary Table S2 lists the reagents and kits used in this work and their catalog numbers.

### Immobilizing targets to the beads

Targets (peptides and domains) were immobilized to azide magnetic beads purchased from Click Chemistry Tools (Scottsdale, Arizona, USA) via a DBCO-S-S-NHS ester linker purchased from Conju-Probe (San Diego, California, USA). These target immobilized beads were used for selection and for subsequent binding tests by the enriched libraries. Target–linker conjugations and bead immobilizations were confirmed via HPLC analysis. For further details of the immobilization protocol and HPLC analyses, refer to the supplementary methods.

### Selection workflows

The SELEX workflow used in this study was designed to run using a KingFisher Flex™ bead particle processor ensuring consistent sample handling throughout the process. In general, selection was carried out for 6 rounds with reduced library and target input combined with increased washing steps in each new round to maintain stringent selection conditions. Prior to selection, the library was heated and quickly cooled to aid in structure formation prior to incubation with the targets. Round 1 was started with approximately $4\times 10^{12}$ unique species for each target. Amplification of the bound pools was performed using gel electrophoresis every few cycles to check the enrichment products in order to minimize overamplification. After amplification, the PCR products were cleaned up on the KingFisher Flex™ to remove excess primers. Strand separation to isolate required biotinylated single strand after amplification was performed in an automated fashion using the azide magnetic beads and DBCO anti-sense primer. For more details on the SELEX process including sample preparation, refer to the supplementary methods.

### Sequencing

Sequencing was carried out for all libraries together in a multiplexed fashion, each sequence set was tagged with a unique predefined index primer obtained from Illumina (San Diego, USA). Sequencing reactions were prepared following the protocol provided with the Illumina 50 cycle sequencing kit and performed on a NextSeq 550 instrument (Illumina). The sequencing results from each enriched or unenriched library were compared pairwise to check for repetitions of the same species within different libraries to confirm no potential cross contamination between them.

### Binding tests

To gauge binding of the enriched libraries to the different targets, biotinylated enriched libraries were incubated with bead immobilized targets followed by washing. The amount of library DNA bound to each target was determined by binding Streptavidin–Horse Radish Peroxidase (HRP) to the biotinylated DNA sequences, then adding HRP substrate, 3,3′,5,5′ tetramethylbenzidine (TMB). 50 ng of library was incubated with 5 μL of target-loaded magnetic beads for 30 minutes with gentle mixing, followed by washing three times in binding buffer to remove unbound DNA, and then by incubation with Streptavidin–HRP for 15 minutes. After three washes in binding buffer, the beads were incubated in the TMB substrate for 15 minutes and the reaction was quenched by the addition of sulfuric acid to a final concentration of 0.1M. The absorbance at 450 nm was recorded.

### Sequence model—training and holdout datasets

Sequencing results from all samples in this study comprised approximately 66.5 million unique sequences. For training purposes, only sequences of 35 nucleotide length were used. All sequences shorter than 35 nucleotides were excluded and all sequences over that length were truncated. The training dataset was comprised of 500 000 randomly sampled sequences from each enriched or unenriched library for a total of 10.4 million sequences (Supplementary Table S3). There were no labels associated with the sequences with regards to which enriched or unenriched library they came from; the masked language model training was thus unsupervised with respect to target or replicate. The model training was also performed with no knowledge of copy number for each sequence (all sequences in the training were unique). Additionally, a holdout dataset was also created consisting of the remaining ∼53 million sequences which were used for validating the models since the trained model has never seen these sequences. All reported analysis results (clustering, KDE overlap, classifier model performance AUC) were performed exclusively with sequences randomly selected from these holdout sequences (see below). The pre-training was performed by masking 15% of the nucleotides across the sequences and then predicting these masked tokens and checking accuracy via backpropagation, enabling the model to learn contextual information about the sequence space provided. No information about the specific target associated with each sequence was provided (the training was unsupervised with respect to label). The learned weights from this pre-trained model were then used as an embedder to generate 2-dimensional latent space representations for any input sequence. Details of the architecture used for this pre-training is provided in Supplementary Fig. S1, and additional details on the pre-training process are given in the supplementary methods.

The pre-trained model was used to create vectorized sequence representations of 10 000 randomly selected holdout sequences from each enriched or unenriched library which were then used to create 2-dimensional sequence representations by applying the Uniform Manifold Approximation and Projection (UMAP) package in Python (McInnes *et al*. 2018). In one replicate of the mutant peptide 1 enrichment, there were only 3800 enriched sequences available and thus all sequences were used (the number of holdout sequences used from each enriched and unenriched library is given in Supplementary Table S3). These representations were then plotted using Seaborn and Matplotlib packages. The 2-dimensional representations were also used to calculate overlap between sequences from distinct sets using Kernel Density Estimation (KDE).

In addition, fine-tuned models were trained as binary classifiers to predict target labels for sequences. For fine-tuning, the weights from the pre-trained model were inherited and fixed during the classifier training such that optimization occurs only on the newly learned weights from two additional dense layers. The fine-tuned models were trained using 50 000 sequences from each enriched or unenriched library sampled from the training set. For each library, classifier model performance was validated using 50 000 randomly sampled holdout sequences. In the case of enriched or unenriched libraries with less than 50 000 unique sequences (i.e. one replicate of the mutant peptide 1 enrichment and one replicate of the beads-only enrichment, Supplementary Table 3), all available holdout sequences were used. The significance of the classification results was further explored via permutation testing where the performance of the classification using the true labels is compared to the performance of 10 000 random permutations of the true labels, resulting in a *P*-value. For more details on pre-training and fine-tuning the sequence models, see the Supplementary Materials.

## Results

A schematic of the overall workflow is depicted in Fig. 1. Random single-stranded oligodeoxynucleotide libraries (approximately $4.0\times 10^{12}$ molecules/target initially) were enriched for binding to each of six protein or peptide targets by performing six rounds of SELEX in a high throughput workflow (Fig. 1A). Each enrichment was performed in triplicate starting from a unique unenriched library. Starting unenriched libraries and final enriched libraries (approximately $35\times 10^{9}$ molecules per sample in a normalized pool) were sequenced. A random sampling of unlabeled sequences from all unenriched and enriched libraries (approximately 16% of the sequenced space) were used as the inputs to a masked language model resulting in a pre-trained model that was used for generating latent space representations of both the training sequences and holdout sequences used for validation (Fig. 1B). The latent sequence representations were used in unsupervised clustering analysis and the pre-trained model was fine-tuned for classification of sequences with regards to the specific target they were enriched to bind (Fig. 1C).

### Enrichment targets

Two protein and four peptide targets that span the wild-type or the mutant 2B (R1306Q) variant form of the Von Willebrand Factor (vWF) domain A1, a blood glycoprotein involved in primary hemostasis, were used to explore the target specificity of latent space sequence representations created by the model (Table 1). vWF-A1-WT domain (207 aa) used in this study was previously used for SELEX development of a DNA aptamer (ARC1172) (Huang *et al*. 2009) along with a known point mutant (vWF-A1-2B: R1306Q) associated with increased bleeding due to platelet clearance from the blood (Cooney *et al*. 1991, Ruggeri 2004). Two overlapping 30 amino acid peptide fragments from each domain spanning the mutation site were also used as enrichment targets. The peptides had varying degrees of sequence similarity, either sharing 40% within overlapping peptides or differing only by one residue between wildtype and mutant pairs. By comparing the latent representations of sequences from enriched libraries from these targets, and from unenriched starting libraries, the target-specificity of the information captured in the model latent space representations can be explored.

The 6 targets in Table 1 were conjugated to magnetic beads for the enrichment process and empty linker beads were used as control targets (beads with just the linker attached, but no target). For each target, selection was performed in triplicate (unenriched samples were generated in duplicate). Each replicate was performed using a distinct random starting library (Fig. 1A) and produced enriched pools with no overlapping sequences (Supplementary Table S4). Replicates also involved independent bead preparations, wash steps, PCR amplification batches, and sequencing reactions. While enrichment-specific artifacts vary across independent SELEX runs, patterns across replicate enrichments are more likely to arise from target-dependent functional constraints rather than sequence memorization or noise. A summary of sequence copy numbers from sequencing results from the unenriched and round 6 enriched libraries are given in Supplementary Table S4.

### Binding of enriched sequences to targets

**Figure 2:**
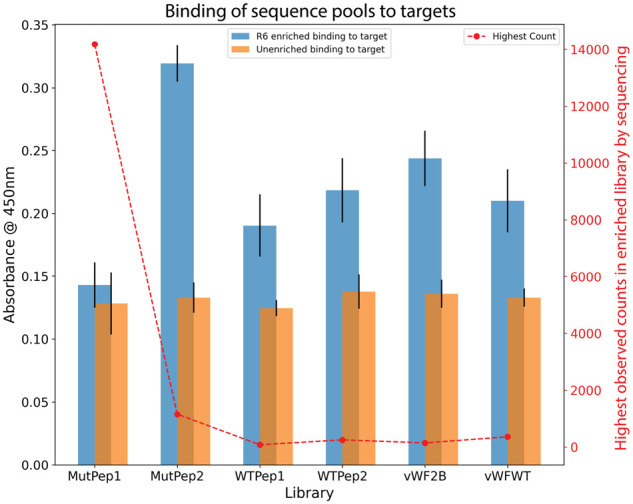
Differential binding to targets between unenriched and enriched libraries. Absorption at 450 nm was used to measure the binding of biotinylated DNA sequences to targets. Average binding across the three enrichment replicates to the targets is shown. The highest sequence copy number observed in libraries is plotted on the secondary *Y*-axis.

Quantitative determination of bound biotinylated library to the targets was performed using streptavidin-HRP detection measuring absorbance at 450 nm. The average absorption of three replicates is shown in Fig. 2. As one might expect, the vWF domain and its point mutant gave statistically similar results. However, the library enriched using wild type peptide 1 (WTPep1, Fig. 2) shows substantially increased binding relative to libraries enriched using mutant peptide 1(MutPep1, Fig. 2), the latter being comparable to binding of unenriched libraries. Both wild type peptide 2 (WTPep2, Fig. 2) and the corresponding point mutant (MutPep2, Fig. 2) resulted in enriched libraries that bound their targets substantially more than unenriched libraries, but the mutant peptide 2 enriched library bound significantly stronger to its target than the WT peptide 2 enriched library did to its target. This difference was consistent across all replicate enrichments (see error bars, Fig. 2). Thus, single amino acid changes in the peptide sequences appear to result in changes in binding of the enriched libraries.

### Sequence based modeling

As described in the methods section, the masked language model used training sequences for both the pre-training and to train binary classifiers, but all of the results shown in the analysis below were performed using the holdout sequences that were never seen by the model. Refer to the Methods and Supplementary Materials for more details.

### Unsupervised analysis of the latent space sequence representations

**Figure 3:**
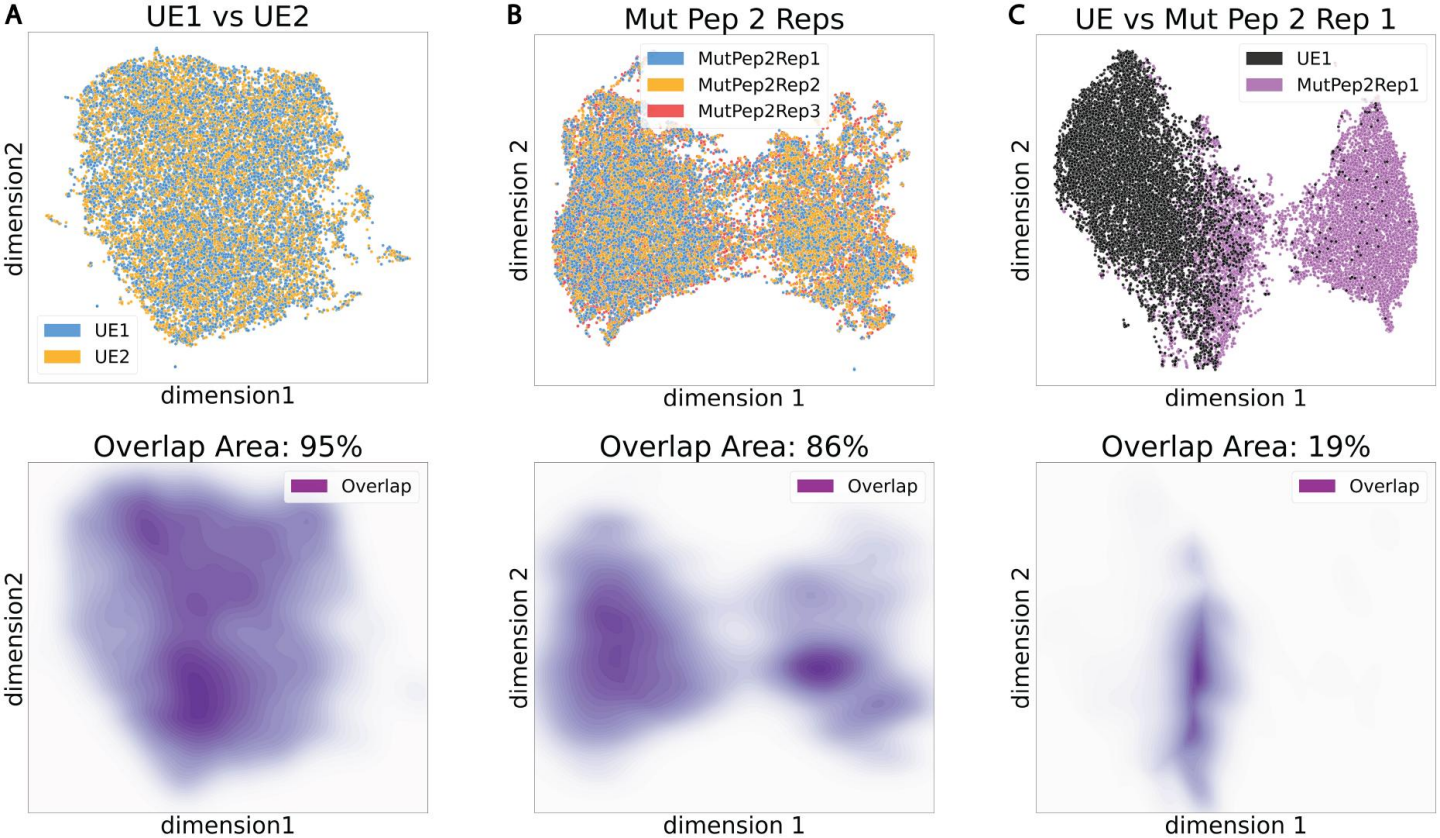
UMAP visualization of sequence latent space representations of hold-out sequences from selected enriched and unenriched libraries. (Top Row) Latent space representations comparing (A) two unenriched libraries, (B) three separate replicate enrichments against mutant peptide 2, and (C) unenriched vs enriched library against mutant peptide 2. (Bottom Row) Kernel density estimate (KDE) plots and overlap values for each comparison.

Figure 3 depicts a comparison of unsupervised clustering of three pairs of libraries. Dimension reduction was performed using UMAP. Kernel Density Estimates (KDE) of distinct sequence sets were then calculated from the 2-dimensional UMAP representations to quantify the degree of overlap of the sequence latent space for different samples. Note that the UMAP dimension reduced representations were used for overlap calculation rather than the high dimensional latent space representation to take advantage of UMAP’s nonlinear ability to condense the information that defines specific clusters into a small number of values, making changes in density more pronounced. The overlap between two completely distinct unenriched libraries was calculated to be 95%, showing almost complete overlap. This high degree of overlap was also observed between three independent replicate enrichments (sharing no common sequences) using mutant peptide 2 as the target with calculated KDE overlap of 87%. This high degree of overlap was observed between all replicate enriched and unenriched libraries in the study (Supplementary Figs. S2 and S3). Note also that UMAP representations of enriched sequences using mutant peptide 2 as the target, which shows strong target binding (Fig. 2), overlap by only approximately 20% with UMAP representations of unenriched sequences; as one might expect, strong binding entails a significant change in information content of the latent space representations.

**Figure 4:**
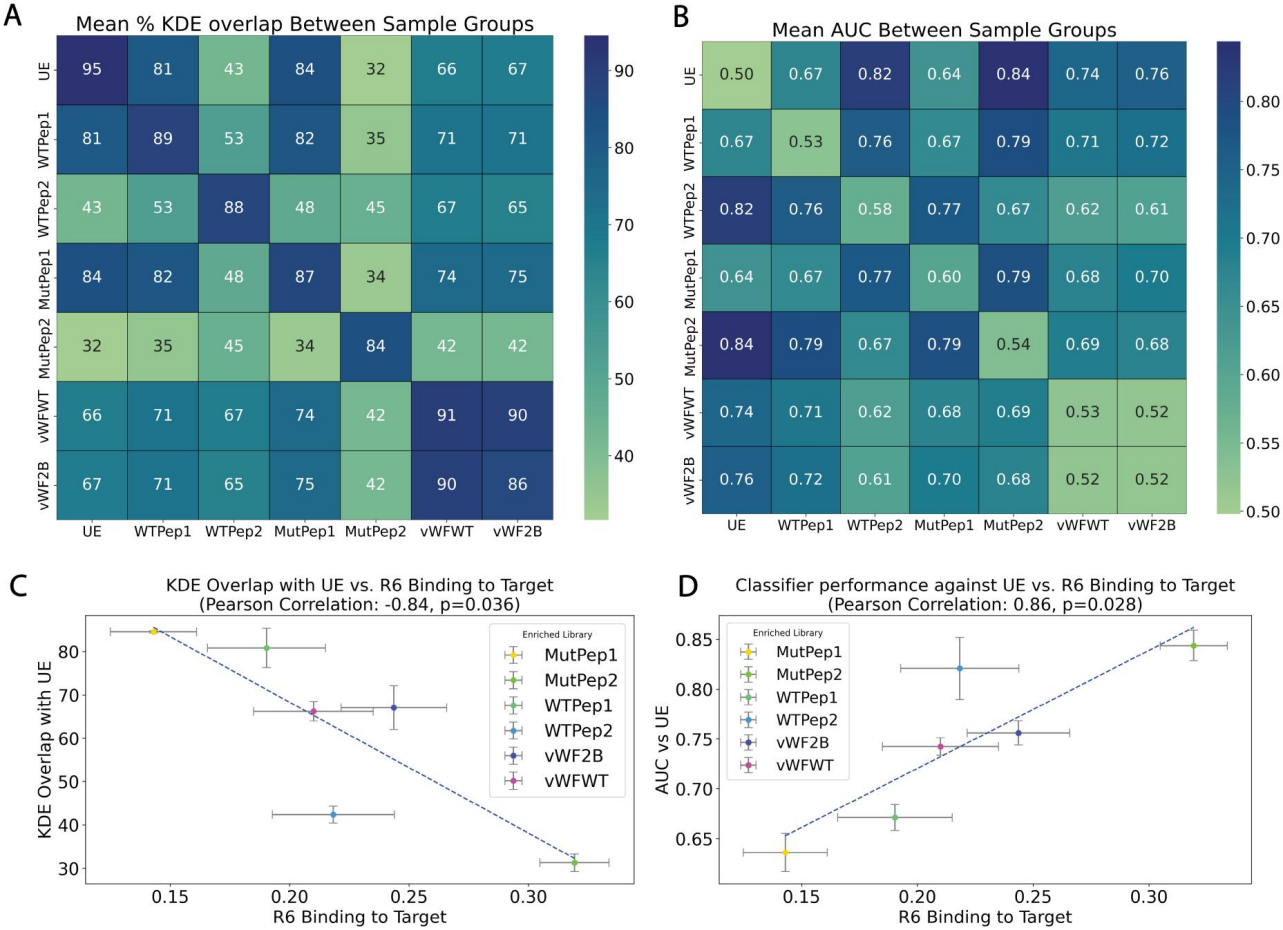
KDE overlap and AUC correlates with binding. (A) Mean KDE overlap values (%) between latent space representations of holdout sequences from each enriched pool comparing each possible pair of targets and with replicates on the diagonal. (B) Mean Area Under the Curve (AUC) from binary classification comparing sequences from each possible pair of targets and with replicates on the diagonal. (C) Linear fit of KDE overlaps vs binding for all possible comparisons between enriched targets and unenriched sequences. Standard deviations across the replicates were calculated and all found to be less than 6%. (D) Linear fit of AUC vs binding for binary classification of each enriched library vs. an unenriched library. Standard deviations across replicates from all comparisons were less than 0.04.

Figure 4A shows the KDE overlap results for all possible library comparisons using different targets. In general, overlap is very high between replicates (diagonal) but quite variable when comparing libraries from different targets. The strong agreement between replicate enriched or unenriched libraries, despite their independent origins, indicates that latent representations reflect consistent target-driven constraints rather than replicate-specific artifacts. The variable KDE overlap between each target’s library and unenriched sequences correlates strongly and inversely (Pearson correlation coefficient r=−0.86, Fig. 4C) with the binding (Fig. 2), implying that libraries that have the least overlap in sequence latent space representation with unenriched libraries also bind their targets most effectively. Thus, sequence latent space representations contain binding information. The error bars for overlap and binding are generated by comparing replicate enrichment sequence data for the targets. Additional visual comparisons of latent space representations between sequence sets from different targets or replicate enrichments are given in Supplementary Figs. S4–S7.

### Binary classification between sequence libraries using different targets

Another approach to determining the specificity of the models trained on sequences enriched for binding to specific targets is to perform binary classification between different target enriched libraries or between a target enriched library and an unenriched library. The pre-trained model was modified by removing the time distributed dense layer from the original masked language model architecture (Supplementary Fig. S8) and then adding two dense hidden layers. Training between two sequence sets involved randomly selecting 50 000 unique sequences from each set without considering copy numbers obtained from sequencing, ensuring a diverse representation of each population. The sampled sequences were then split into training/test sets with an 80/20 ratio, respectively. Performance of the test set during the training was compared to performance of 50 000 holdout sequences using the same trained classifier. Classifier performances were reported as area under the curve (AUC) for the holdout set. We observed almost identical performance between test sequences and the holdout sequences, confirming that the model did not significantly overfit the data (Supplementary Table S5).

The results for all possible binary classification pairs using holdout sequences are shown in Fig. 4B. AUCs between unenriched libraries were 0.50 indicating no ability to classify the labels. This was also observed when using two simulated unenriched libraries (random sequences generated in silico) (Supplementary Fig. S9). AUCs between target replicate enriched pools (diagonal) are also near 0.50. Classification between enriched samples is mixed, ranging from 0.52 to 0.84. AUC values between pairs are anticorrelated with KDE overlaps, as can be seen comparing Fig. 4A and B. A scatter plot comparing KDE overlap and classifier AUC values is given in Supplementary Fig. S10, and the Pearson correlation coefficient is −0.97. As with KDE overlap, AUC correlates strongly with binding when comparing enriched and unenriched libraries (Fig. 4C and D) with a Pearson correlation coefficient of 0.86 (compared to a −0.84 for KDE overlap). To assess the statistical significance of the classifier performances, permutation testing was performed. True labels in the classification were permuted 10 000 times and compared to classifier results to determine the number of times the permuted labels resulted in a classification performance either comparable or higher than non-permuted labels and plotted as a distribution. All classifications between non-replicate libraries were statistically different from random label comparisons, having *P* values below 0.05 (Supplementary Fig. S11 for more details and examples) indicating that the observed classifier performances were not due to random chance.

**Figure 5:**
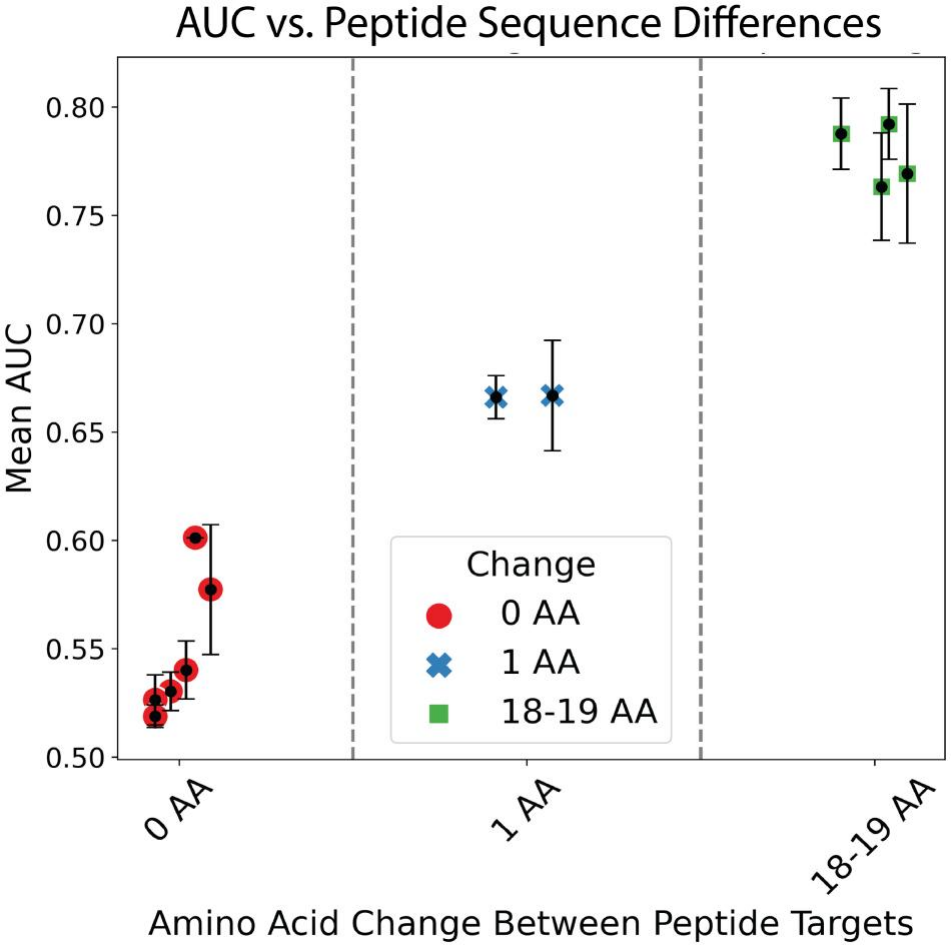
AUC values from classification between enriched libraries for three categories of sequence overlap between targets: complete overlap, 1/30 amino acid changes, and 18–19/30 amino acid changes.

The variation in AUC between enriched libraries also varies systematically with target similarity among the peptides. AUC values fall into three groups depending on the similarity of the targets of enrichment (Fig. 5). Replicates (complete similarity) have the lowest AUC values near 0.50. Single amino acid changes between target peptides move the value to about 0.67. When the 30 residue peptides overlap by 11–12 amino acids (18–19 amino acids different), the AUC ranges from 0.76−0.79.

### Discriminating information content as a function of copy number in the library

**Figure 6:**
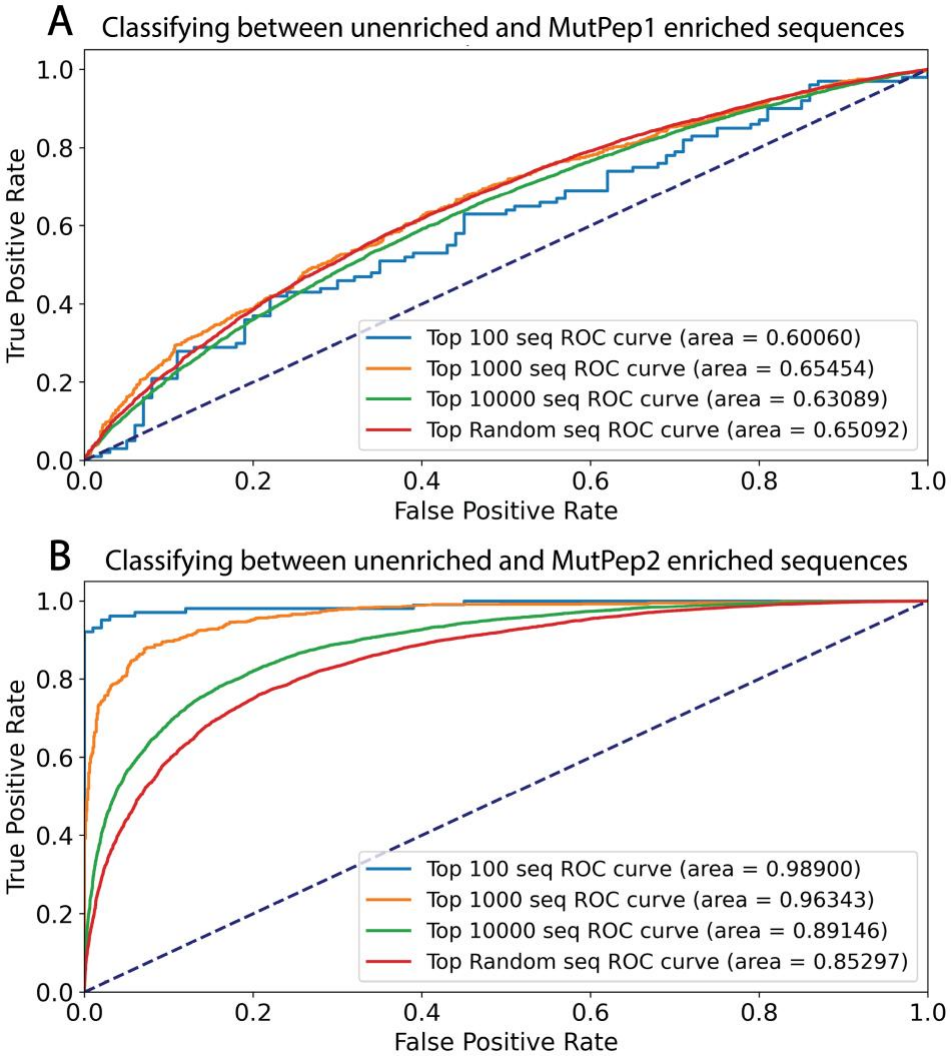
Receiver operator curves (ROC) resulting from variable sampling of sequences via copy numbers. (A) Classification comparing mutant peptide 2 enriched and unenriched sequences when sampling sequences of varying copy numbers. (B) Classification comparing mutant peptide 1 enriched and unenriched sequences when sampling sequences of varying copy number.

Fine-tuned models were also trained as classifiers using sequences sampled based on the number of copies in the libraries enriched using mutant peptides 1 and 2 as targets. Mutant peptide 1 enriched libraries show very little binding to the target while Mutant peptide 2 enriched libraries bind their targets strongly (Fig. 1). Samples compared included the sequences with the highest 10 000, 1000 and 100 copy numbers in the enriched sequence library and AUC values were determined relative to unenriched sequences. While there is effectively no consistent relationship between copy number and binding (Fig. 1, Supplementary Table S4), an increase in AUC was observed with increasing copy number in mutant peptide 2 enriched sequences. However, there was no copy number dependence on classification with enrichments generated using mutant peptide 1 (Fig. 6). The variable relationship between classification performance, copy numbers and binding was also observed in the other enrichments against the wildtype peptides (Supplementary Table S6). For example, while enriched pools against the vWF domains showed reasonable performance in classification from unenriched pools, the top 100 species by copy numbers from sequencing were found to not perform as well as the top 1k or 10k sequences. In fact, random sampling of sequences resulted in the best classification. Apparently, while the copy number of specific sequences is correlated with increased information content in some enrichment cases, this is not generally the case.

## Discussion

This study demonstrates that it is possible to use machine learning approaches to train a general encoder capable of producing latent DNA sequence representations that quantitatively capture the evolution of information content during enrichment for binding to multiple targets. DNA sequence libraries were enriched against six related targets in triplicate each: wildtype and point mutant A1 domains of vWF, two overlapping peptide regions (30mer) from each domain spanning the mutated residue. The binding of the resulting enriched libraries to their targets was variable, but consistent between enrichment replicates (Fig. 2) even though they all contained completely unique sequences. One of the mutant peptide targets produced enriched libraries with little capacity to bind. This was not observed in the wild-type variant of the peptide target. Sequences randomly sampled from across all the enriched libraries were combined and used to train a bidirectional Long Short-Term Memory model using a Masked Language Model approach. The resulting model weights were subsequently used as an embedding both to generate latent space representations of sequences for unsupervised analysis and as the basis for fine tuning classifiers to distinguish enriched samples from each other or from unenriched libraries.

It is possible to employ other more traditional approaches for pattern recognition such as k-mer clustering of the sequences themselves. As described in supplementary materials, one can see general trends using local sequence models of that nature. However, particularly with regard to the depth of information content, as characterized by the ability to separate enrichments to different targets in an unsupervised manner, local sequence models are limited, as shown in supplementary figure S13 where UMAP representations like Fig. 3 of unsupervised analysis are compared between a k-mer representation (KDE overlap = 0.81) and an MLM representation (KDE overlap = 0.40) as used in Fig. 3. One advantage of the MLM approach, which may be important in the comparison of figure S13, is that they are able to consider interactions across the entire nucleic acid sequence, providing a more robust representation of possible structures arising from enrichement. Moreover, training the MLM is general and largely unbiased, not requiring specific parameter selections like k-mer size or number of clusters, and it requires only a few GPU-hours for training, offering no substantial computational disadvantage compared to other approaches.

### Independent replicate enriched and unenriched libraries result in essentially indistinguishable outcomes

Unsupervised clustering of latent space representations of sequences from independent enrichment replicates overlapped quantitatively (Fig. 3B and Fig. 4A) indicating that the information content of the latent space representation is quite reproducible in enrichment to the same target starting from completely independent initial libraries and enrichments with no sequence overlap. This is also reflected in the classification results comparing replicates to one another (Fig. 4B); the enriched library replicates were effectively indistinguishable even with supervised training. Because replicate enrichments were carried out independently (including distinct random starting libraries, independent bead preparations, separate PCR amplification, and independent sequencing), the consistency of latent representations across replicates indicates that the model learned target-dependent constraints rather than enrichment-specific artifacts. This conclusion is further supported by the ability to distinguish targets including, to a limited extent, differing by only a single amino acid (Fig. 5) and by the strong correlation between latent-space divergence and experimental binding (Fig. 4C and Fig. 4D). All analyses were performed using holdout sequences not used in masked language model training, confirming that the observed structure reflects functional enrichment rather than memorization.

### Binding is correlated with unsupervised clustering of latent space sequence representations and with classification accuracy

One of the most significant outcomes of this study, as alluded to above, is the finding that the binding for the targets used is directly correlated with both the overlap of unsupervised clustering and the classification accuracy of supervised analysis between enriched and unenriched libraries (Fig. 4C and Fig. 4D). Clustering overlap and classification accuracy are also highly correlated (Supplementary Fig. S10). The implication of this, borne out in the analysis of sequence samples not involved in the training of the original general model or the fine-tuned classifiers at all, is that a substantial difference in the latent space representation of enriched vs. unenriched sequences is consistent with significant binding to a target. While there could be differences that arise due to artifacts like binding to the substrate the targets are anchored to or unusually easily copied sequences in the original pool, differential information content between the enriched and unenriched sequences is a necessary condition for binding and thus useful as an indicator. Additionally, the correlation suggests that it should be possible in some cases to predict the composition of sequences that have yet higher binding values.

### Copy number is not a general metric for binding potential of an enriched library to its target

Variable levels of specific sequence enrichment, as gauged by sequence copy number, was observed in enriched libraries across the different targets (Supplementary Table S4). Cluster separation in latent space and high classification performance between enriched and unenriched pools (Fig. 4) was more indicative of target binding than was sequence copy number in an enriched library.

### Specificity of binding is also captured in the latent space representations and classifier models

Binding affinity is only part of what one would hope to capture in a predictive enrichment model. Just as important is the ability to predict specificity of binding. As shown in Fig. 5, there are statistically significant differences in the classifier accuracy in considering three different well defined target comparisons: replicates using the same peptide target, comparison between a peptide and a single mutant of that peptide and comparison between two peptides that overlap in sequence by 11-12 amino acids (differ by 18-19 amino acids). At least in these cases, there is discernable differentiation between sequences as similar as one amino acid change in 30, implying that this level of specificity can be embedded in the latent space representation and learned by subsequent fine tuning to train a classifier. Fine discrimination between targets with significant similarity further supports the concept that the model is not simply learning general enrichment noise.

### Predictive power of sequence-based models for SELEX enrichments

One of the most interesting conclusions from this work, both from a fundamental and practical perspective, is that machine learning models trained on sequence sets that aggregate enriched sequences from multiple distinct targets can learn what constitutes an enriched sequence, at least within the context of that set of targets. This suggests that there are common structural constructs in enriched sequence libraries among multiple targets, in agreement with past work (Iwano *et al*. 2022, Sun *et al*. 2022, Lee *et al*. 2023). Furthermore, the model demonstrates potential for identifying and characterizing these motifs, for example through an iterative process of sequence design, model evaluation, sequence modification, and structural modeling of optimized sequence classes. Fundamentally, this paves the way for studies investigating the mechanisms of enrichment from an information and structural perspective. At a practical level, it has the potential to allow more effective initial library design and more rapid optimization of protocols to achieve desired affinity and specificity of target binding.

## Supplementary Material

vbag118_Supplementary_Data

## Data Availability

The datasets and code used in the study are available on Zenodo: https://doi.org/10.5281/zenodo.14941815.
