# Supplementary material for: From randomness to recognition: modeling the evolution of DNA sequence information during enrichment for binding
Source: Bioinform Adv. 2026 Apr 26;6(1):vbag118. doi: 10.1093/bioadv/vbag118 (PMC13291818; doi:10.1093/bioadv/vbag118)
Supplement: vbag118_Supplementary_Data [file vbag118_Supplementary_Data.zip › 26-May-2026_054543_Supplementary_file.pdf]

From Randomness to Recognition: Modeling the Evolution of DNA Sequence Information During Enrichment for Binding

Varun Maher[1,2,3], Daniel Martin[1], David Spetzler[1], Zhan-Gong Zhao[1], Heather O'Neill[1] and Neal W. Woodbury[2,3,*]

[1] Caris Life Sciences, Precision Medicine Target and Drug Discovery, Tempe, Arizona, United States of America

[2]Arizona State University, School of Molecular Sciences, Tempe, Arizona, United States of America

[3]Center for Molecular Design and Biomimetics, Biodesign Institute, Arizona State University, Tempe, Arizona, United States of America

*Corresponding author: nwoodbury@asu.edu

Availability and Implementation: The datasets and code used in the study are available on Zenodo: https://doi.org/10.5281/zenodo.14941815

Supplementary Section

Supplementary Methods

Target immobilization to magnetic azide beads

Peptide and protein domain targets were immobilized on azide magnetic beads using a DBCO-S-S-NHS ester. The disulfide in the linker was reduced with TCEP to gently elute the DNA bound only to the target. For coupling, an NHS ester on the linker is reacted first with N-terminal primary amines on the targets by incubating 48 nmoles of target with 24 nmoles of acetonitrile reconstituted linker in 100 µL of 1X PBS at pH 7.2 at room temperature and shaking at 500 RPM for 4 hours. After the incubation, the ester–primary amine reaction was quenched by addition of 1M Tris-HCl to a final concentration of 0.1M and then cooling on ice for 30 min. The quenched solution was then added to PBS-washed azide beads followed by gentle mixing. The total volume was then increased to 200 µL using additional 1X PBS. This was then mixed on a shaker at 500 RPM overnight at 4 °C. After incubation, the magnetic beads were removed with a magnet, and the supernatant was stored for HPLC analysis. The target immobilized beads were washed 3 times with 1X PBS, 0.05% pluronic F-127 and then stored in 200 µL at 4°C.

HPLC analysis of conjugated and immobilized samples

All HPLC runs were performed using a Thermofisher Scientific (Waltham, MA, USA) Vanquish autosampler instrument using a 250 X 2.1 (mm) Acclaim™ Vanquish C18 column, with the column and column pre-heater set to 60 °C.

The HPLC gradient used is depicted in **Supplementary Table S7**, and **Supplementary Fig. S12** contains histograms showing target-linker conjugated peaks and absence of these peaks in the supernatant of immobilization reactions with azide beads.

Selection Workflows

Selection Protocol

Selection workflows were carried out using a KingFisher Flex bead processor from ThermoFisher Scientific (Waltham, MA, USA). All KingFisher Flex instrument protocol files can be provided upon request. The sample plates (unenriched single stranded DNA library in 1X PBS, 5mM $MgCl_2$, 0.05% pluronic F-127), wash solutions (1X PBS, 5 mM $MgCl_2$, 0.05% pluronic F-127), and elution solution (10 mM TCEP) were all loaded on the instrument prior to running. The target loaded beads were incubated with 200 ng ssDNA library for 1 hour to start Round 1 (4 x 10^{12} unique species), followed by three washes for three minutes each and eluted at room temperature for 10 minutes. Each progressive round of selection saw a 10% reduction in starting library yield used and a one-minute increase in the mixing time of the beads per wash step. In round 4 two additional wash plates were introduced to increase selection stringency. The entire elution volume was used in the PCR reaction described after selection. PCR amplifications were checked every few cycles for amplification intensity, to reduce the possibility of off-size amplicons, and some samples were amplified for more cycles than others to obtain enough amplified product.

Sample preparation for SELEX round

The starting library was mixed with the required amount of 10X PBS (1X final), 1M $MgCl_2$ (final 5mM) and water, mixed well and then heated to 95 °C for 5 minutes followed by rapid cooling to 4 °C for 10 minutes. This was then mixed with yeast tRNA and sheared salmon sperm DNA as competitor at a final concentration of 125 ng/µL each, along with pluronic F-127 at a final concentration of 0.05%.

PCR amplification

PCR amplification was carried out using Q5 Hi fidelity hot start Taq-Polymerase (New England Biosciences) and the sample buffer provided. The PCR mix was incubated for 30 seconds at 98 °C, followed by cycling as follows: 30 seconds at 98 °C, followed by 30 seconds at 60 °C, followed by 1 minute at 72 °C. After all cycles, the reaction was held at 72 °C for 3 minutes followed by holding at 4 °C.

Biotin strand recovery

After the PCR was complete, magnetic azide beads were used to capture the DBCO-tagged double stranded amplified DNA, then subjected to denaturing conditions to release the biotinylated strand using a KingFisher system to perform the bead handling. Excess primers and reducing agent were first removed from the PCR reaction using silica beads and a chaotropic binding (NTI buffer) step followed by an ethanol-based wash (NT3 buffer). NTI buffer is a guanidinium isothiocyanate buffer that is used in DNA purification systems to promote DNA binding to silica supports. NT3 buffer is an ethanol-based buffer that helps with washing away excessive salts and other impurities from the sample after binding to silica support. Both buffers were obtained from Macherey-Nagel (Düren, Nordrhein-Wesfalen, Germany). The magnetic silica bead cleanup was followed by capturing the double stranded product with azide beads. The azide beads were first washed in 5X PBS, then incubated with cleaned double stranded DNA in the presence of 5X PBS at 42 °C overnight. This incubation was followed by two 5-minute washes in 1 mL water each followed by a 20 second wash in 3 mM NaOH at room temperature to wash away any non-specifically bound double stranded material. Then a 10-minute elution in 100 µL of 30 mM NaOH at 56 °C was performed. The

elution was quenched with 10 μL of 300 mM HCl and incubated at 4 °C for 15 minutes with shaking at 500 RPM.

Binding tests

Binding of biotinylated enriched libraries to targets on beads was quantified using the reaction of Streptavidin – Horse Radish Peroxidase (HRP) to the biotinylated libraries followed by reacting HRP with 3,3',5,5' tetramethylbenzidine (TMB) substrate. Libraries (50 ng) were incubated with 5 μL of target-loaded magnetic beads in 1X PBS, 0.05% pluronic F-127 for 30 minutes at 500 RPM, followed by washing 3X with 1X PBS, 0.05% pluronic F-127 for 1 minute each at room temperature shaking at 500 RPM. The washed library bound beads were incubated at room temperature with streptavidin-HRP for 15 minutes followed by 3X washing using the same mixing conditions. Then the washed beads were incubated in TMB substrate for 15 minutes followed by quenching by addition of sulfuric acid to a 0.1M final concentration. Absorbance was then read at 450nm.

Sequence model

Sequencing results from all samples in this study comprised approximately 66.5 million unique sequences. For training purposes, we only used sequences of 35n length. All sequences shorter than 35n were dropped and all sequences over that length were trimmed. The training dataset was assembled comprised of randomly sampled sequences from each enrichment target consisting of a total of 10.4 million sequences (S2 Table). Additionally, a holdout dataset was also created consisting of the remaining ~ 53 million sequences. The training dataset was used to train a Masked Language Model (MLM), using only sequence information, with no copy number data. The architecture used for the pre-training step is provided in S1 Fig. Fifteen percent of the nucleotides across the tokenized sequences were masked and the MLM was trained to predict the masked tokens. This enabled self-supervised contextual learning about the sequence space provided. The model was built using tensorflow and keras libraries in python. The MLM used a biLSTM architecture, utilizing 256 LSTM units and the pre-training itself was performed for 500 epochs.

The model architecture and weights were saved and used both to create latent space sequence representations for UMAP analysis and to serve as the functional model in the fine-tuning process as well. In most cases, only a portion of holdout sequences were used to validate model performance; 10,000 sequences were sampled randomly from each label for UMAP visualization and 50,000 sequences were sampled randomly from each for the classifiers. For a few enrichments (Supplementary Table S3) there were not enough sequences, in which case all holdout sequences were used.

<u>Creating UMAP visualizations</u>

The pre-trained model was used to create latent space representations of the 10,000 holdout sequences from each label. These vectorized representations were used as the basis for dimension reduction using the UMAP package in python (parameters: n_components = 2, n_neighbor = 15, min_dist = 0.1 and used the 'correlation' metric). The resulting 2 dimensional coordinates from the UMAP output were plotted using the Seaborn and Matplotlib libraries in python. The overlap between sequence sets was determined using Kernel Density Estimation (KDE) using the Gaussian KDE class packaged within the SciPy library in Python. All reported KDE overlaps in this study utilized only holdout sequences.

<u>Comparing latent space representations from a MLM model to representations from a KMER model</u>

The training sequences described for model training above were also used to train a KMER model using CountVectorizer and a KMER length of 4. This resulting model was again used to create a UMAP 2-dimensional representation. One such comparison between all replicates of enriched sequences using wildtype peptide 2 as a target and unenriched sequences is shown in **Supplementary Fig S11**.

<u>Fine-tuning models on target labels/Testing trained classifiers on holdout data</u>

Classifier training/fine-tuning was performed using the keras library in python. Classifiers were trained with the same sequences used in creating the pre-trained model, but the performance of each classifier was determined using holdout sequences to rule out any overfitting. To train a classifier, the weights from the MLM were inherited from the pre-trained model and fixed such that the only newly learned weights were

from the last two additional dense layers (**S8 Fig**). An 80:20 train/test split was used for training. All possible unique classifier combinations between the 20 sequence sets (190 unique pairs) were trained. Fifty-thousand sequences sampled randomly from each label in the holdout sets were used to validate the generalization capabilities of the fine-tuned models by comparing performance to the test sequences. The classifier performances were recorded as area under the curve (AUC). All reported AUCs in this study are with holdout sequences. The original labels were permuted 10,000 times and these new permuted distributions were compared to the predicted distributions of the original labels to obtain a p-value by calculating the number of permuted distributions that had an AUC equal or greater to the observed (unpermuted) AUC:

$$p - value = \frac{\sum (permuted\ AUCs\ \geq observed\ AUC)}{n\ permutations}$$

Supplementary Figures and Tables

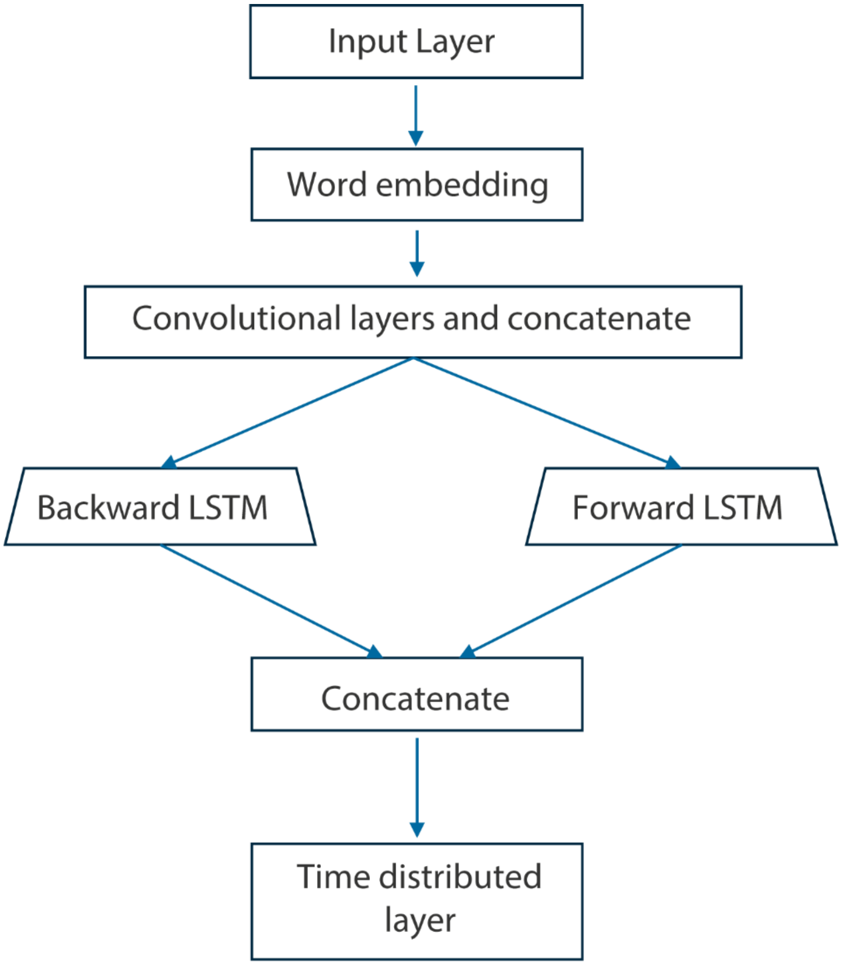

Supplementary Figure 1. Simple illustration of pre-training architecture. Layer descriptions provided below:

InputLayer – Sequence input layer

Embedding – This layer converts input sequences into dense vectors of fixed size where each token in the sequence is represented as an embedded vector

Convolutional layers: Used to capture local patterns in the data and then concatenated

LSTM layers: Long-term sequence dependencies are captured using a biLSTM architecture with 256 units each

Concatenate: LSTM layer outputs are merged to combine the learned features

Time distributed layer: Fully connected dense layer to each time step, performing a classification on the learned features (semi-supervised)

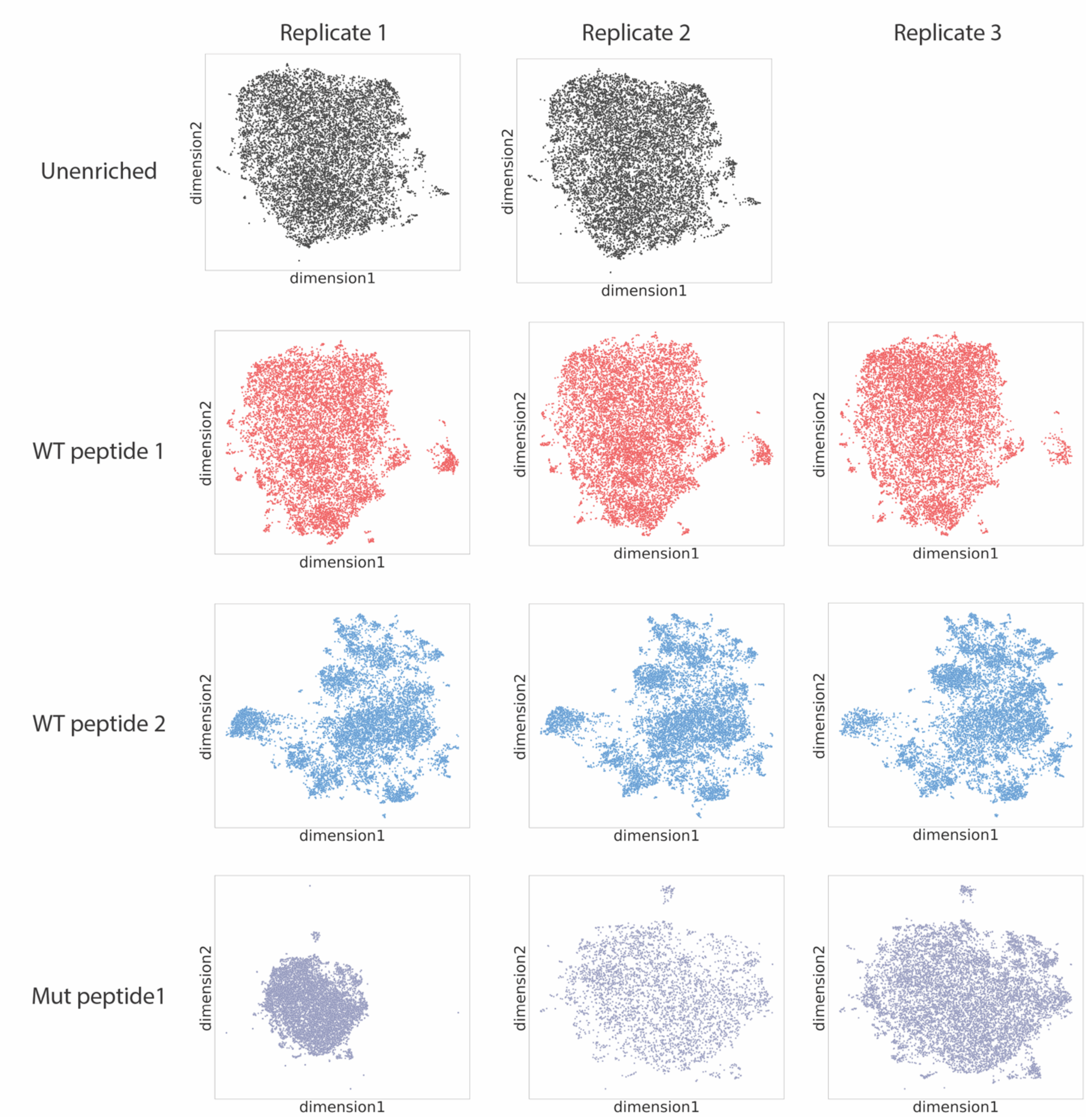

Supplementary Figure 2. Similar UMAP representations between all enrichment replicate pools in latent space. These distributions in latent space are determined using the pre-trained model generated using label free holdout sequences not seen before by the model

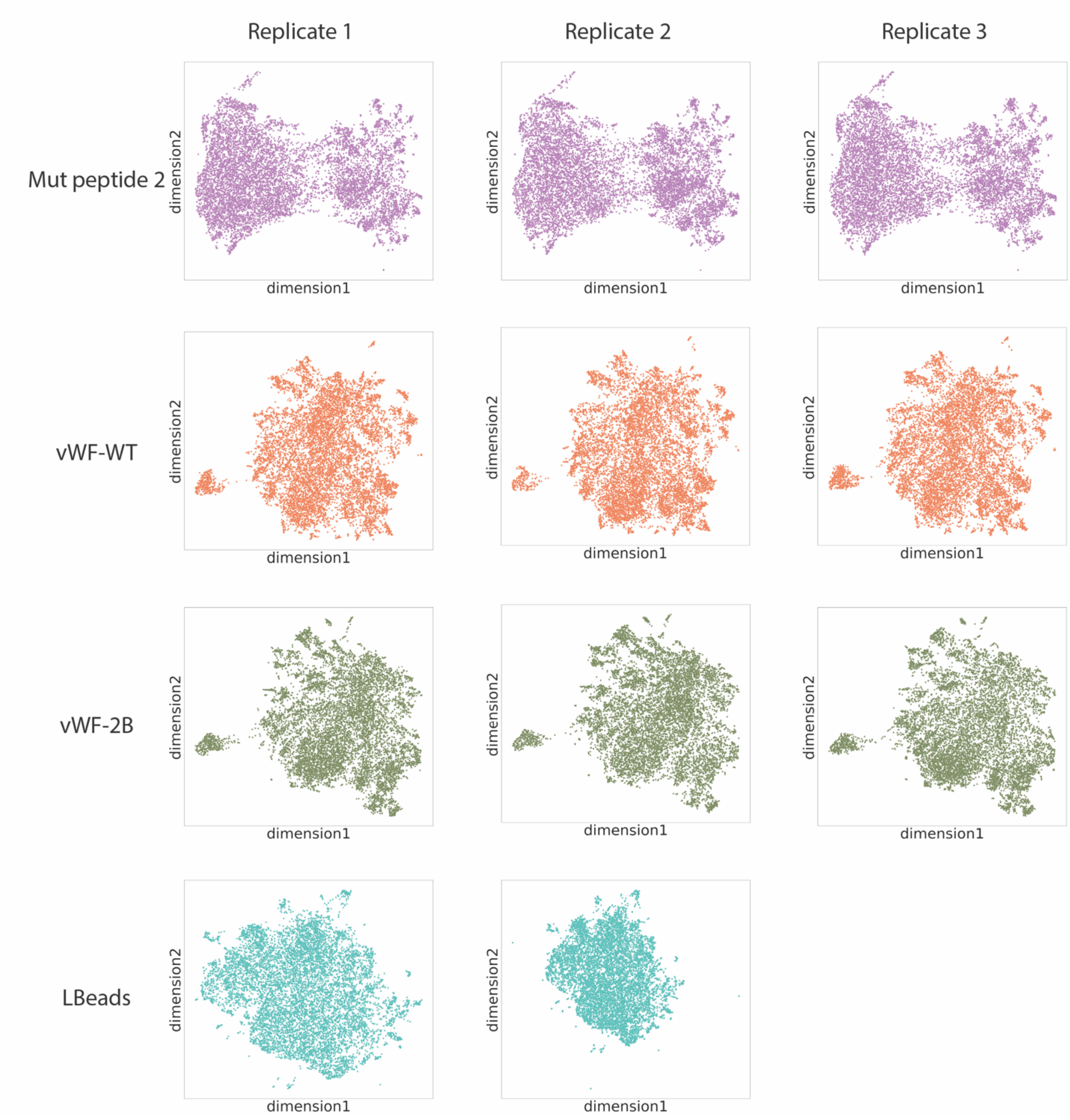

Supplementary Figure 3. Similar UMAP representations between all enrichment replicate pools in latent space. These distributions in latent space are determined using the pre-trained model generated using label free holdout sequences not seen before by the model

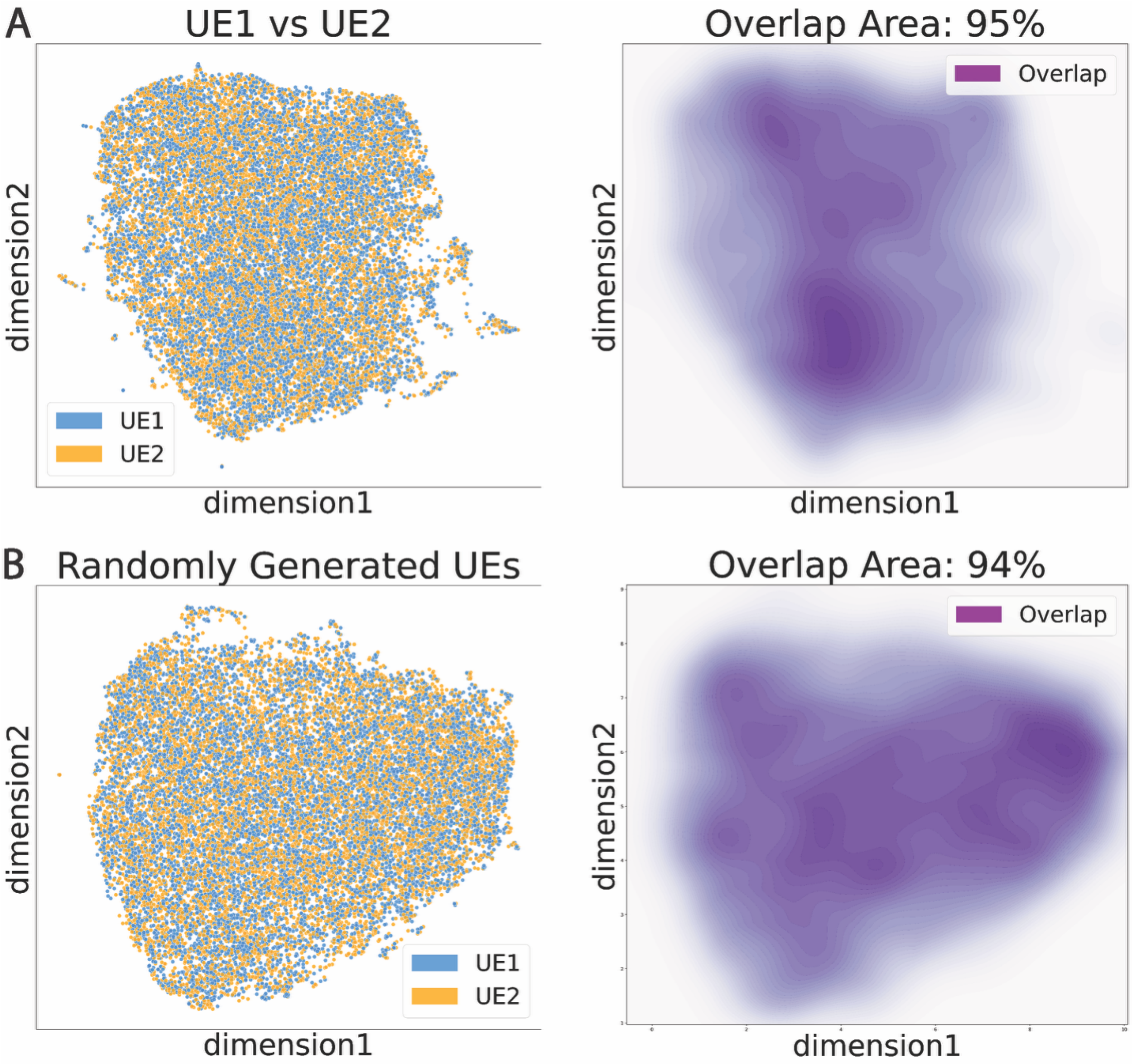

Supplementary Figure 4. Latent space representations of unenriched sequences. Representations of holdout sequences in the left column with KDE overlap plots on the right.

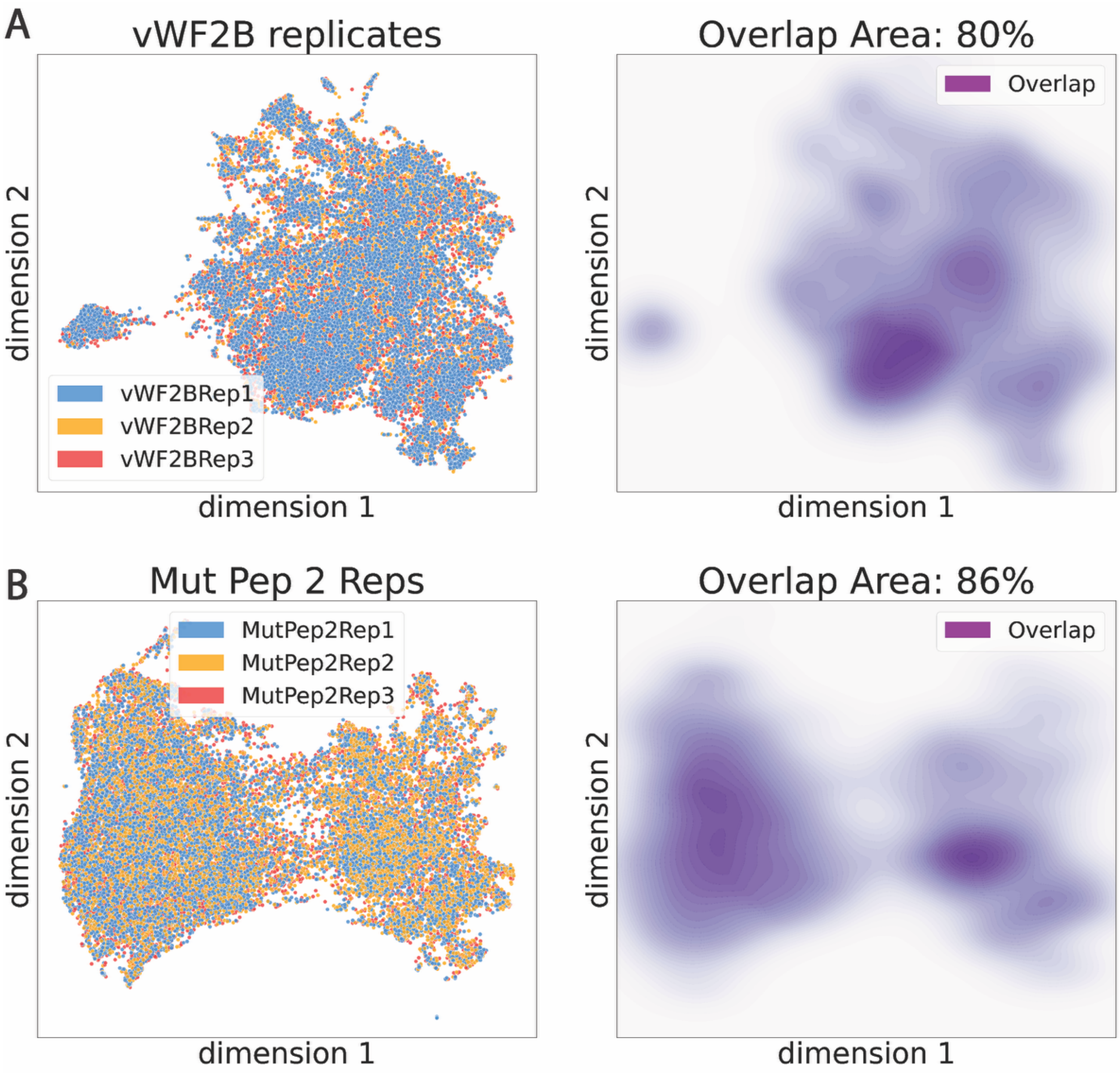

Supplementary Figure 5. Latent space representations of replicate enrichments. Representations of the holdout sequences in the left column with KDE overlap plots on the right.

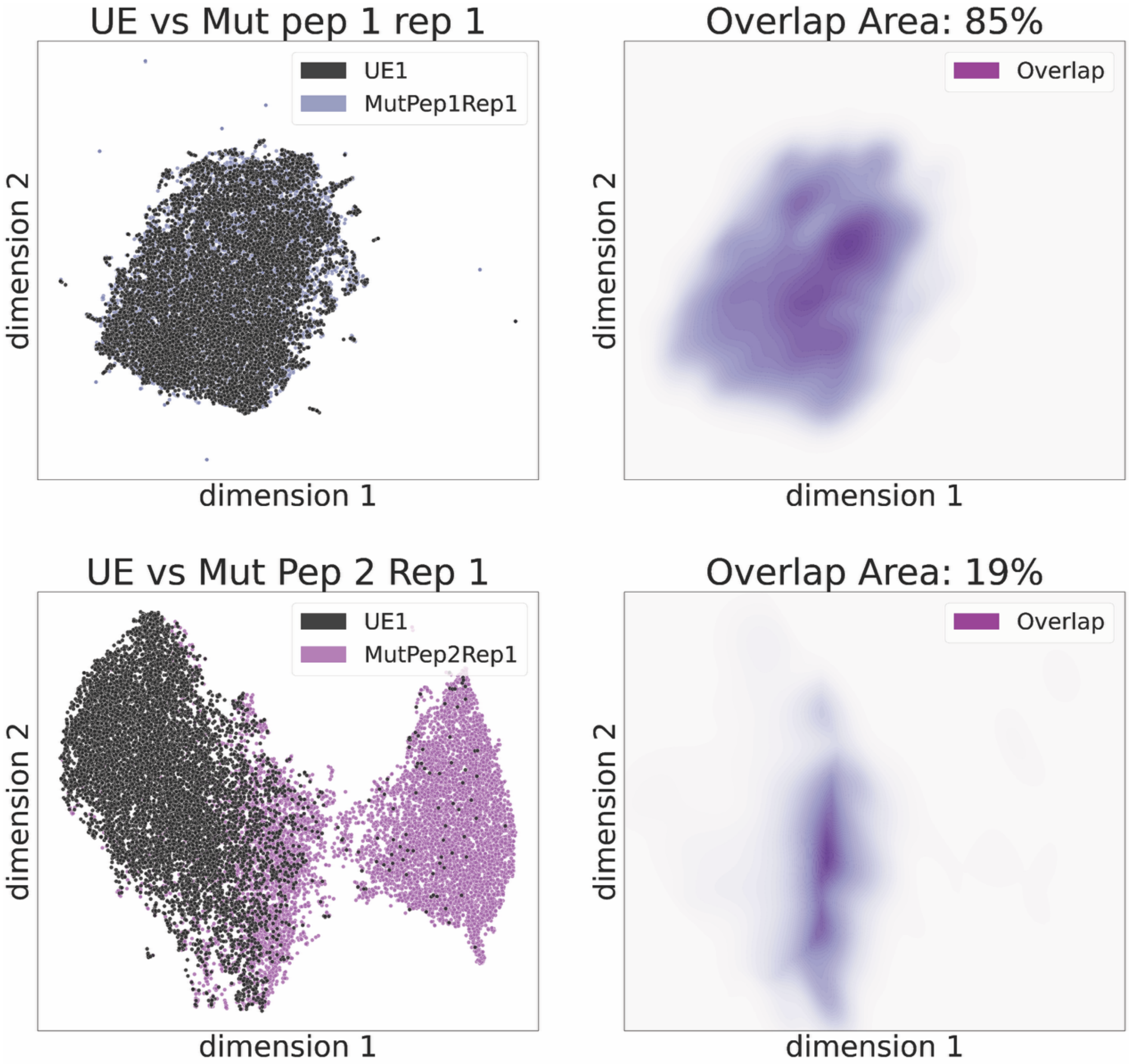

Supplementary Figure 6. Latent space representations comparing unenriched and enriched sequences. Representations of the holdout sequences in the left column with KDE overlap plots on the right.

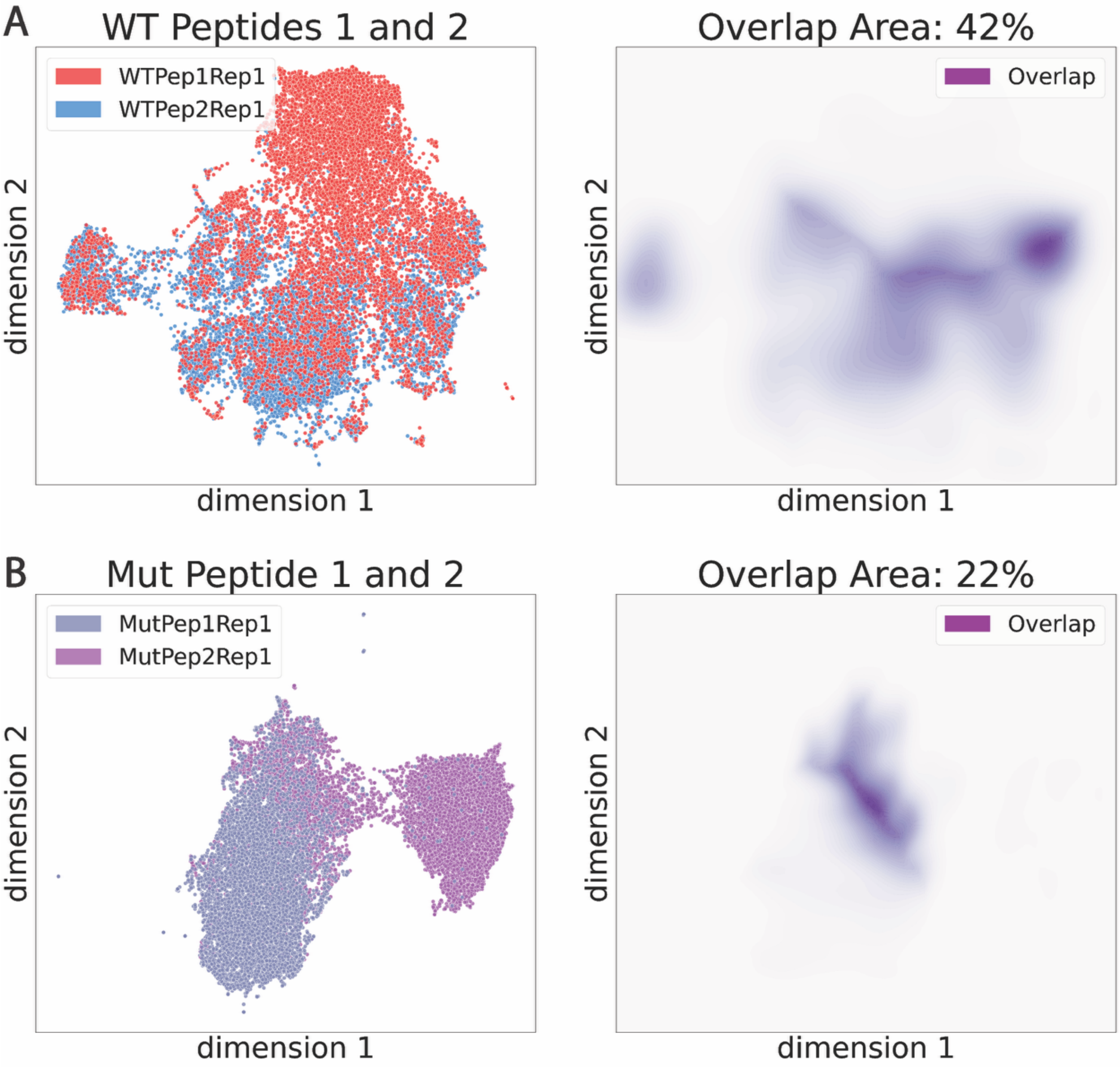

Supplementary Figure 7. Latent space representations comparing enriched sequences. Representations of the holdout sequences in the left column with KDE overlap plots on the right.

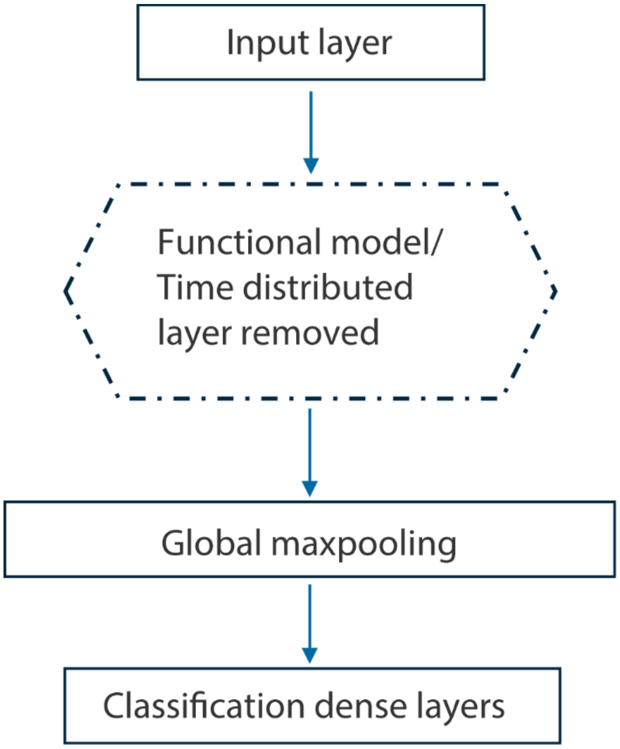

Supplementary Figure 8. Fine-tuning architecture for training on selection target label. Layer descriptions provided below:

InputLayer – Sequence input layer

Functional – The entire pre-trained model architecture from S1 FigA with time distributed layer removed. Output is 512-dimensional feature map

Global_max_pooling1d: Reduces feature map to its maximum value across entire sequence, distilling most prominent features from a sequence

Dense layers: Fully connected layers responsible for binary classification

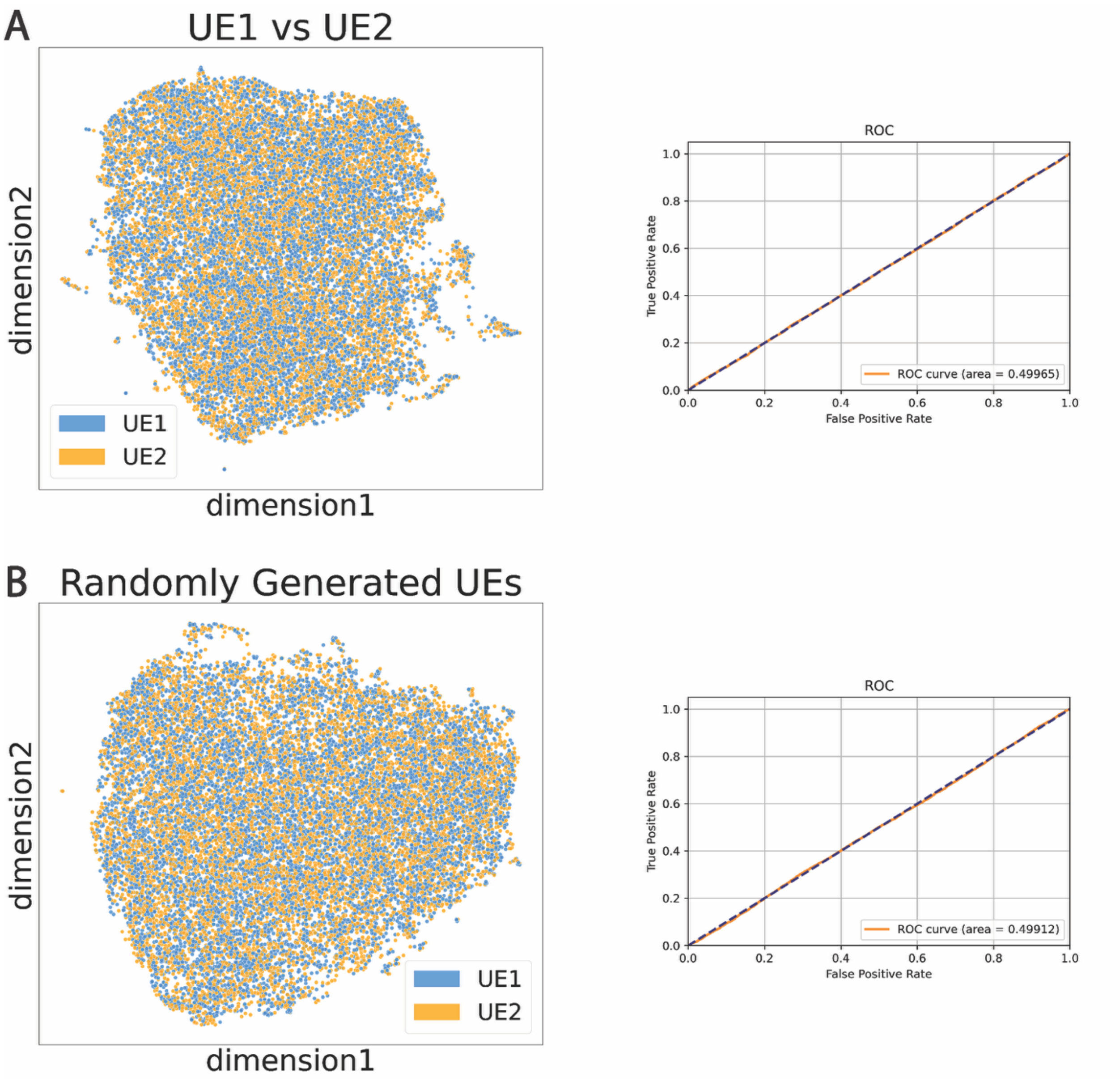

Supplementary Figure 9. Latent space representations and classification accuracy comparisons between experimentally sequenced unenriched libraries and simulated unenriched libraries. There is no obvious unsupervised clustering in these examples and classification with fine-tuning is random.

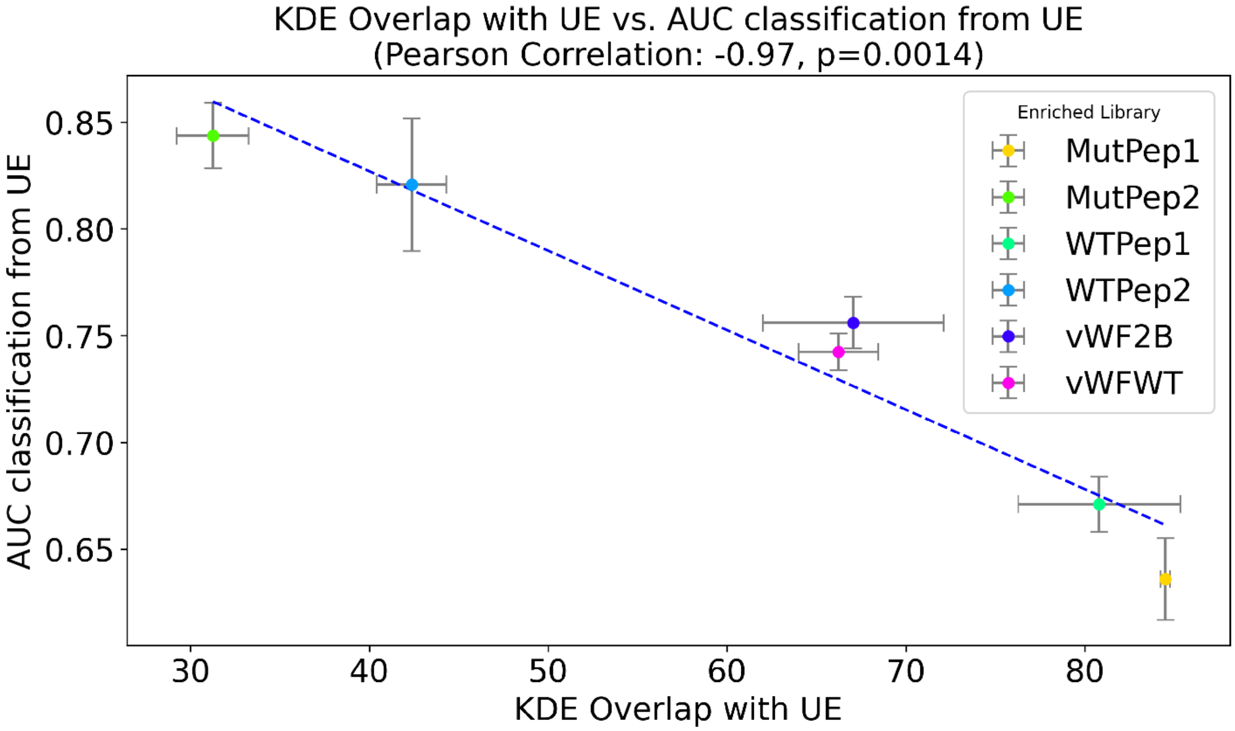

Supplementary Figure 10. Linear relationship between KDE overlaps and classification AUC between sequence sets. Plot showing the linear relationship between KDE overlaps and classification AUCs when comparing unenriched and enriched sequence sets. The data was found to have an inverse but highly linear relationship, suggesting that classification accuracies can be inferred strongly using latent space overlaps between the sequence sets. The standard error points are derived from latent space overlaps and classification AUCs between unenriched and all three enrichment replicates.

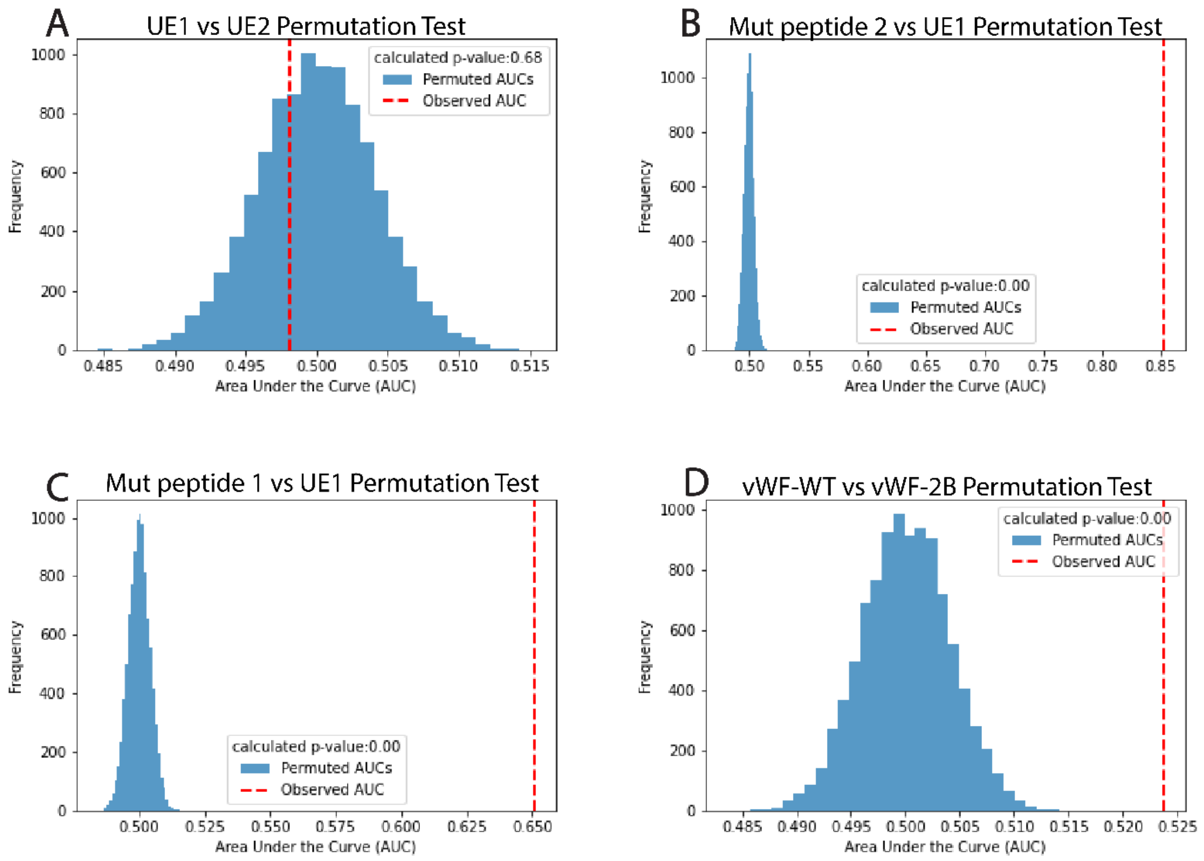

Supplementary Figure 11. Permutation tests of the significance of trained classifier results – (A) Permutation test resulted in a p-value of 0.68, indicating a result that can be obtained by chance, something expected for classifying between two unenriched sequence sets. With the introduction of target enriched libraries in classification vs unenriched sequences, the p-value drops below 0.05 for all classifications **(B-D)**, even in the case of enrichments that produced high-copy number libraries that did not bind to their targets, and that did not classify in comparisons with unenriched sequences.

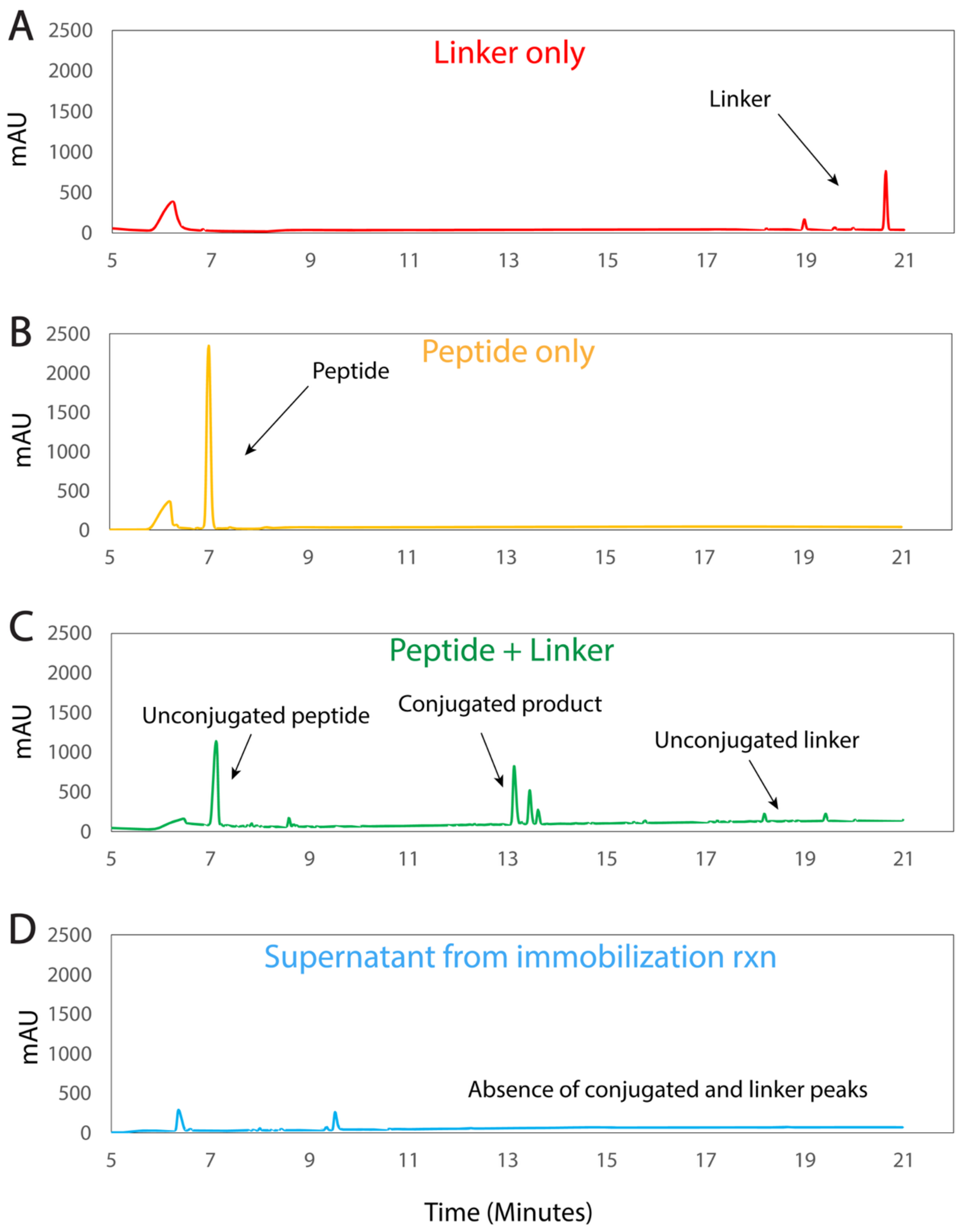

Supplementary Figure 12. HPLC runs verifying conjugations and target immobilization to beads. HPLC chromatograms showing the absorbance of **(A)** Linker used for conjugations, **(B)** A peptide target by itself, **(C)** Conjugation reaction between a peptide target and linker showing unreacted linker and peptide with conjugated product in the middle and **(D)** Immobilization supernatant showing absence of conjugation and linker peaks confirming attachment to the beads.

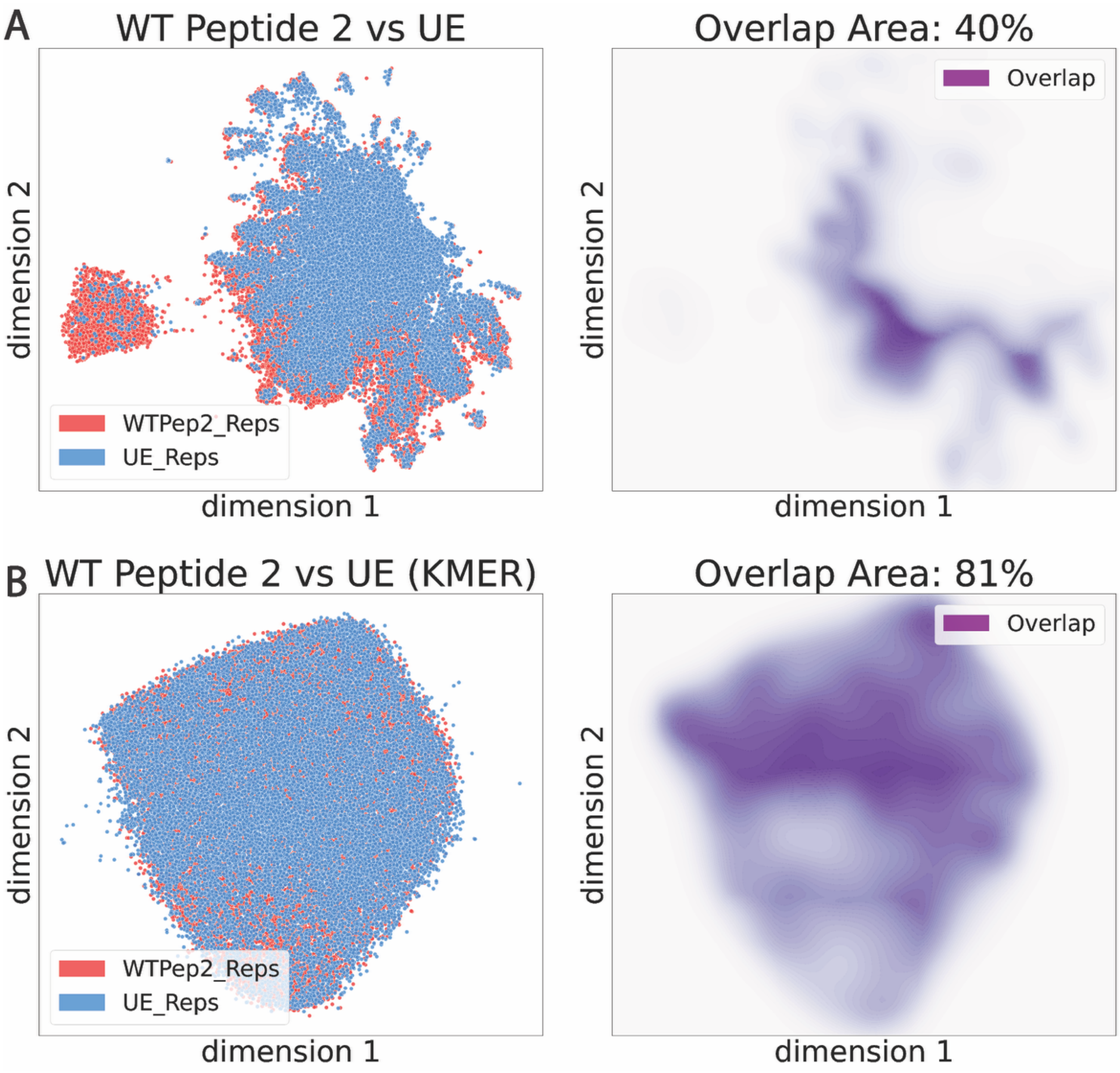

Supplementary Figure 11. Latent space representations derived using a KMER model. In the case of some enriched libraries, separation as seen using the biLSTM model (**A**) is lost when using a KMER model (**B**).

Oligonucleotide	Sequence
UE 35n	/5Biosg/AGTCGATGTCACTCGATACG(N:25252525)(N)(N)(N)(N)(N)(N)(N)(N)(N)(N)(N)(N)(N)(N)(N)(N)(N)(N)(N)(N)(N)(N)(N)(N)(N)(N)(N)(N)(N)(N)(N)(N)(N)(N)(N)CTGTCTCTTATACACATCTGACGCTGCCGACGA/3Phos/
UE 35n+1	/5Biosg/AGTCGATGTCACTCGATACG(N:25252525)(N)(N)(N)(N)(N)(N)(N)(N)(N)(N)(N)(N)(N)(N)(N)(N)(N)(N)(N)(N)(N)(N)(N)(N)(N)(N)(N)(N)(N)(N)(N)(N)(N)(N)(N)ACTGTCTCTTATACACATCTGACGCTGCCGACGA/3Phos/
UE35n+2	/5Biosg/AGTCGATGTCACTCGATACG(N:25252525)(N)(N)(N)(N)(N)(N)(N)(N)(N)(N)(N)(N)(N)(N)(N)(N)(N)(N)(N)(N)(N)(N)(N)(N)(N)(N)(N)(N)(N)(N)(N)(N)(N)(N)(N)GACTGTCTCTTATACACATCTGACGCTGCCGACGA/3Phos/
UE 35n+3	/5Biosg/AGTCGATGTCACTCGATACG(N:25252525)(N)(N)(N)(N)(N)(N)(N)(N)(N)(N)(N)(N)(N)(N)(N)(N)(N)(N)(N)(N)(N)(N)(N)(N)(N)(N)(N)(N)(N)(N)(N)(N)(N)(N)(N)TGACTGTCTCTTATACACATCTGACGCTGCCGACGA/3Phos/
Sense Primer	/5Biosg/AGTCGATGTCACTCGATACG
Anti-sense Primer	/5DBCON//iSp18/AAAAAAAAAAAAAAAAAAAAAAAAAAAAAA/iSp18/TCGTCGGCAGCGTCA

Supplementary Table 1. Oligonucleotide details. Oligonucleotide orders were placed through IDT (Integrated DNA Technologies – Coralville, IA) via Ultramer synthesis. The 35n region was designed to maintain an equal representation of the 4 nucleotides. Four different syntheses of unenriched sequences were mixed in equimolar amounts to form the master unenriched library used in the study.

Supplementary Table 2. List of reagents and consumables used in experimental workflows with supplier and catalog information provided.

Product	Supplier	Catalog #
1M Tris HCl, pH 8.0	Thermofisher Scientific	15586-025
PBS Salt	Sigma-Aldrich	P3813
10% Pluronic F-127	Thermofisher Scientific	P6866
10% Tween-20	Sigma-Aldrich	P9416
1M MgCl2	Thermofisher Scientific	AM9530G
Acetonitrile	Fisher chemical	A461-4
Trifluoroacetic acid	Thermofisher Scientific	28904
0.5M TCEP	Thermofisher Scientific	77720
Sheared Salmon Sperm DNA	Thermofisher Scientific	1563-011
Yeast tRNA	Thermofisher Scientific	AM7119
Q5 Hi-Fidelity Hot Start PCR Kit	NEB	M0493L
100mM dNTPs	Thermofisher Scientific	10297117
10M NaOH	Sigma-Aldrich	72068
Sulfuric acid	Thermofisher Scientific	039669.K2
Formic acid	Sigma-Aldrich	33015
TMB Substrate	Thermofisher Scientific	34028
4% EX Agarose Pre-cast gel	Thermofisher Scientific	G401004
Azide beads	Click-Chemistry Tools	1036-5
Silica beads (PureBind M Beads)	Ocean-nanotech	P0202-050
DBCO-SS-NHS ester linker	Conju-Probe	CP-2024
NTI buffer	Macherey-Nagel	740305.120
NTC buffer	Macherey-Nagel	740654.100
NT3 buffer	Machery-Nagel	740598
Qubit DS DNA HS kit	Thermofisher Scientific	Q32854
Sequencing kit (50 cycle, SBS)	Illumina	20024906
NextSeq Flowcell	Illumina	2022408
250 X 2.1 mm Acclaim Vanquish C18 column	Thermofisher Scientific	074812-V

Supplementary Table 3. Number of sequences used in training the pre-trained model and the number of sequences remaining in the holdout sets.

Sequence Set	# sequences in train	# sequences in holdout
WT pep 1 -1	5.00E+05	1.27E+06
WT pep 1 -2	5.00E+05	3.49E+06
WT pep 1 -3	5.00E+05	4.06E+06
WT pep 2 -1	5.00E+05	1.66E+06
WT pep 2 -2	5.00E+05	5.58E+06
WT pep 2 -3	5.00E+05	3.96E+06
Mut pep 1 -1	5.00E+05	5.87E+04
Mut pep 1 -2	1.00E+05	3.83E+03
Mut pep 1 -3	5.00E+05	3.46E+05
Mut pep 2 -1	5.00E+05	2.14E+06
Mut pep 2 -2	5.00E+05	2.06E+06
Mut pep 2 -3	5.00E+05	1.02E+06
vWF-WT -1	5.00E+05	1.81E+06
vWF-WT -2	5.00E+05	1.81E+06
vWF-WT -3	5.00E+05	9.02E+05
vWF-2B -1	5.00E+05	1.03E+06
vWF-2B -2	5.00E+05	1.33E+06
vWF-2B -3	5.00E+05	1.83E+06
Lbeads -1	4.00E+05	8.57E+04
Lbeads -3	4.00E+05	3.39E+04
UE-1	5.00E+05	7.75E+06
UE-2	5.00E+05	1.14E+07
Total	1.04E+07	5.36E+07

Supplementary Table 4. Results for 22 sequence sets are displayed showing total number of sequences, total number of unique sequences, and the results for the numbers of species at or above certain numbers.

	Species with counts						
Sample	>=1	1	>1	>50	>100	>500	>1000
UE1	8.29E+06	8.20E+06	9.18E+04	0	0	0	0
UE2	1.20E+07	1.18E+07	1.48E+05	0	0	0	0
WT Peptide 1 - 1	1.80E+06	1.39E+06	4.13E+05	0	0	0	0
WT Peptide 1 - 2	4.20E+06	2.21E+06	1.99E+06	81	5	0	0
WT Peptide 1 - 3	4.81E+06	2.39E+06	2.41E+06	185	19	0	0
WT Peptide 2 - 1	2.33E+06	1.17E+06	1.16E+06	14262	901	0	0
WT Peptide 2 - 2	6.27E+06	3.70E+06	2.57E+06	0	0	0	0
WT Peptide 2 - 3	4.71E+06	2.01E+06	2.69E+06	3	0	0	0
Mut Peptide 1-1	5.65E+05	3.80E+05	1.85E+05	33113	21597	5196	1703
Mut Peptide 1-2	1.04E+05	6.11E+04	4.30E+04	5554	2186	17	1
Mut Peptide 1-3	8.77E+05	5.55E+05	3.22E+05	54298	31642	2357	129
Mut Peptide 2-1	2.84E+06	1.36E+06	1.48E+06	12089	2378	15	0
Mut Peptide 2-2	2.71E+06	1.32E+06	1.39E+06	12804	2964	34	2
Mut Peptide 2-3	1.65E+06	8.21E+05	8.25E+05	31274	9224	168	12
vWF_WT-1	2.50E+06	1.12E+06	1.37E+06	2188	44	0	0
vWF_WT-2	2.46E+06	1.06E+06	1.39E+06	1464	16	0	0
vWF_WT-3	1.51E+06	8.09E+05	7.03E+05	32476	3765	0	0
vWF_2B-1	1.56E+06	7.36E+05	8.20E+05	196	3	0	0
vWF_2B-2	1.90E+06	8.56E+05	1.04E+06	2602	84	0	0
vWF_2B-3	2.51E+06	1.10E+06	1.41E+06	2247	43	0	0
LBeads-1	4.92E+05	3.29E+05	1.63E+05	42443	31096	1624	73
LBeads-3	4.40E+05	3.05E+05	1.35E+05	26799	23120	8050	1845

Supplementary Table 5. Classifier performances reported as AUCs between test and holdout sequences. Main results are reported using holdout sequences.

Comparing performance of holdout sequences to test sequences in binary classifier		
	Test Set	Holdout Set
Sample	avg AUC vs UE	avg AUC vs UE
WT Peptide 1	0.67	0.67
WT Peptide 2	0.82	0.79
Mut Peptide 1	0.64	0.63
Mut Peptide 2	0.84	0.84
vWF-WT	0.74	0.74
vWF-2B	0.76	0.75

Supplementary Table 6. Variable classifier performance observed with sequence sampling via copy numbers across the different enrichments. Variable performances were similar between replicates.

	AUC vs unenriched			
Target	Random	Top 10k	Top 1k	Top 100
WT Peptide 1	0.67	0.71	0.77	0.81
WT Peptide 2	0.82	0.85	0.92	0.95
Mut Peptide 1	0.64	0.64	0.66	0.66
Mut Peptide 2	0.84	0.89	0.97	1.00
vWF-A1-WT	0.74	0.74	0.72	0.69
vWF-A1-2B	0.76	0.76	0.72	0.68

Supplementary Table 7. HPLC gradient to test target – linker conjugations and bead immobilizations.

Time (min)	Flow (mL/min)	%A	%B
0	0.4	95	5
3	0.4	95	5
13	0.4	65	35
33	0.4	55	45
36	0.4	20	80
37	0.4	0	100
40	0.4	0	100
41	0.4	95	5
46	Stop Run		

Buffer A: 99.9% H_2O, 0.1% Formic acid, 0.02% Trifluoroacetic acid
Buffer B: 99.9% acetonitrile, 0.1% Formic acid, 0.02% Trifluoroacetic acid
